# Sequence-based genome-wide association study reveals genetic and metabolic mechanisms underlying feed efficiency-related traits in beef cattle

**DOI:** 10.1186/s40104-025-01341-3

**Published:** 2026-01-27

**Authors:** Leonardo M. Arikawa, Lucio F. M. Mota, Larissa F. S. Fonseca, Gerardo A. Fernandes Júnior, Bruna M. Salatta, Gabriela B. Frezarim, Patricia I. Schmidt, Sindy L. C. Nasner, Julia P. S. Valente, Amalia M. Pelaez, Roberta C. Canesin, Josineudson A. II. V. Silva, Maria Eugênia Z. Mercadante, Lucia G. Albuquerque

**Affiliations:** 1 São Paulo State University (UNESP), School of Agricultural and Veterinarian Sciences, Jaboticabal, SP 14884-900 Brazil; 2 Acaraú Valley State University (UVA), Sobral, CE 62040-370 Brazil; 3 National Council for Science and Technological Development, Brasilia, DF 71605-001 Brazil; 4 Beef Cattle Research Center, Institute of Animal Science, Sertãozinho, SP 14174-000 Brazil; 5 Cia de Melhoramento, PRO – Produção Profissional, São José Do Rio Preto, SP 15061-580 Brazil; 6 São Paulo State University (UNESP), School of Veterinary Medicine and Animal Science, Botucatu, SP 18618-681 Brazil

**Keywords:** *Bos indicus*, Energy metabolism, Imputed WGS, Muscle development, Oxidative phosphorylation, Residual feed intake

## Abstract

**Background:**

Efficiency is characterized by maximum productivity with lower inputs and minimal waste, resulting in greater output with the same or even fewer resources. In livestock, more efficient animals in converting food into protein may improve the economic efficiency of production systems, as feed costs represent a significant expense in beef production. Thus, the present study aimed to use imputed whole-genome sequencing (WGS) data to perform a genome-wide association study (GWAS) in order to identify genomic regions and potential candidate genes involved in the biological processes and metabolic pathways associated with feed efficiency-related traits (RFI: residual feed intake, DMI: dry matter intake, FE: feed efficiency, FC: feed conversion, and RWG: residual weight gain) in Nellore cattle.

**Results:**

The GWAS identified significant SNPs associated with feed efficiency traits in Nellore cattle. A total of 42 SNPs were detected for RFI, 10 for DMI, 99 for FC, 15 for FE, and 3 for RWG, distributed in different autosomes. Annotation analysis identified several candidate genes, and the prioritization highlighted 21, 9, 68, 23, and 8 key genes for RFI, DMI, FC, FE, and RWG, respectively. The prioritized candidate genes are involved in muscle development, lipid metabolism, response to oxidative stress, nutrient metabolism, neurotransmission, and oxidative phosphorylation. Additionally, enrichment analysis indicated that these genes act in several signaling pathways related to signal transduction, the nervous system, the endocrine system, energy metabolism, the digestive system, and nutrient metabolism.

**Conclusion:**

The use of imputed WGS data in GWAS analyses enabled the broad identification of regions and candidate genes throughout the genome that regulate expression of feed efficiency-related traits in Nellore cattle. Our results provide new perspectives into the molecular mechanisms underlying feed efficiency in Nellore cattle, offering a genetic basis to guide the breeding of efficient animals, thereby optimizing resource utilization and the profitability of production systems.

**Supplementary Information:**

The online version contains supplementary material available at 10.1186/s40104-025-01341-3.

## Background

Improvements in feed efficiency (FE) of beef cattle have the potential to increase farmers' profitability, as feed costs have a great economic impact on the production system and may account for up to 80% of total costs [1]. Efficiency is characterized by maximum productivity with lower inputs (i.e., intake) and minimum waste, that is, producing more with the same or even fewer resources. In this way, the use of more efficient animals in converting food into protein may improve the profitability of production systems, as the same level of production can be achieved with fewer inputs [1, 2].

Feed efficiency can be improved through feeding and management practices. However, the observed genetic variation in FE-related traits presents a valuable opportunity to enhance animal efficiency through selection by breeding programs [3, 4]. Dry matter intake (DMI) has a significant effect on FE and is a key variable in calculating feed efficiency-related traits such as feed conversion (FC) and residual feed intake (RFI). Feed conversion, defined as the amount of feed consumed per unit of weight gain, has been traditionally used to assess FE. However, FC does not consider the mobilization of body reserves, which may overestimate the efficiency of animals that use these reserves to sustain production [5]. Alternatively, RFI is an FE-indicator trait that has gained popularity in recent decades, as it can reflect intrinsic variations in metabolic processes that influence productive efficiency [6]. Moreover, there is evidence that efficient animals, those with low RFI, produce less enteric methane compared to high-RFI animals [7]. Thus, the selection of efficient animals not only optimizes the use of available resources and reduces feed costs but also contributes to more sustainable beef production.

Despite the increased economic and environmental interest in improving FE, its selection is not carried out frequently in Brazil due to the difficulties and costs associated with measuring data at the individual level. Therefore, molecular information emerges as a promising alternative to reduce dependence on direct phenotypic measurements, although these remain necessary to ensure accurate genetic evaluations, contributing to a more accessible and efficient selection process [8]. Recent advances in genome sequencing technologies have revolutionized global livestock production, allowing the identification of millions of variants across the entire genome [9]. However, despite the substantial reduction in sequencing costs over the last decade, association studies based on sequence-level genotypes are still financially restrictive, especially in emerging countries. In this context, genotype imputation has become a central component of association studies, expanding genomic coverage using data generated by microarrays, which have a significantly lower cost [10]. Thus, imputing SNP arrays at the sequence level offers a more detailed genetic assessment, considering all genotypic variation and enabling a broader discovery of regulatory regions and possible causal variants associated with complex traits [11].

Feed efficiency traits are complex traits, influenced by multiple genes, environmental factors, and the interaction between genetic and these environmental factors. Previous research has been conducted to identify chromosomal regions and genes associated with FE through genome-wide association studies (GWAS), providing insights into the genetic control of these traits [12–17]. However, most of these studies were carried out based on genotypic data derived from SNP panels, which restrict the scope for broadly identifying genome-wide variants and QTL underlying FE. Moreover, the available GWAS studies based on sequence genotypes are predominantly focused on taurine breeds, which may limit the applicability of the findings to indicine breeds due to genetic and adaptive differences between these subspecies. In this context, the present study aimed to use whole-genome sequencing (WGS) data to perform GWAS to identify genomic regions and potential candidate genes involved in the biological processes and metabolic pathways associated with feed efficiency-related traits in Nellore cattle.

## Methods

### Phenotypic data

The data on feed efficiency-related traits were obtained from 2,321 Nellore animals (1,966 males and 355 females), born between 2004 and 2021, progeny of 217 sires and 1,207 dams. The animals belonged to an experimental breeding program at the Institute of Animal Science (Beef Cattle Research Center—Sertãozinho, Brazil, *n* = 2,030) and two commercial Nellore breeding programs of Qualitas (*n* = 116) and Cia do Melhoramento (*n* = 175). The database was composed of information on residual feed intake (RFI), dry matter intake (DMI), feed efficiency (FE), feed conversion (FC), and residual weight gain (RWG). All experimental procedures were performed in live animals, therefore slaughter or euthanasia was not required for data collection.

The animals were subjected to performance tests that occurred for an average period (± standard deviation) of 83.3 ± 13.1-d at the Institute of Animal Science, 56 ± 0-d at Qualitas, and 55.9 ± 3.4-d at Cia do Melhoramento. The animals started the test at an average age of 286 ± 41.1 (Institute of Animal Science), 657.3 ± 36.9 (Qualitas), and 543.4 ± 28.15 (Cia do Melhoramento) days and were kept in individual or collective pens. The collective pens were equipped with electronic feed bunks (GrowSafe^®^, Airdrie-AB, Canada; or Intergado^®^, Contagem-MG, Brazil) for automated recording of individual daily feed intake, with diet and water offered ad libitum. Thus, all animals within each contemporary group (CG) had access to the same feed-intake recording system throughout the test. The feeders were refilled twice a day (8-h and 15-h), and their diet consisted basically of corn silage, *Brachiaria* hay, soybean meal, ground corn, and mineral salt with added urea. In each test, a single diet was offered to animals that varied according to the breeding program, with diets formulated to achieve an average daily gain of 1.1 kg/d for the Institute of Animal Science and Cia do Melhoramento, while Qualitas aimed for a gain of 1.7 kg/d. At the Institute of Animal Science, selection is based on yearling weight, whereas animals from the Qualitas and Cia do Melhoramento breeding programs are selected based on growth, visual scores, and precocity indicators traits.

The feed efficiency-related traits were obtained as described by Benfica et al. [4]. The DMI was obtained as the average of all valid days of feeding intake, adjusted for the dry matter content of each week. The average daily gain (ADG) was determined as the linear regression coefficient of body weight (BW) on days in test (DIT) according to the equation: $y_{i\;}=\;\alpha\;+\;\beta\;\times\;{\mathrm{DIT}}_i\;+\;e_i$, where ${y}_{i}$ is the animal BW in the *i*^th^ observation, $\alpha$ is the intercept corresponding to the initial BW, $\beta$ is the linear regression coefficient corresponding to ADG, and $e_i$ is the random error. The FE and FC traits are two measures of feed efficiency that indicate how well an animal converts the feed it consumes into a useful product; FE is calculated as the ratio of ADG to DMI, while FC is determined by the ratio of DMI to ADG. The mid-test metabolic weight (BW^0.75^) was calculated as: ${\left[\alpha + \, \left(\mathrm{0.5} \, \times \it\text{ DIT }\times \, {\mathrm{ADG}}\right)\right]}^{0.75}$. RFI was calculated as the residual of the linear regression of DMI on ADG and BW^0.75^, according to the equation: $\mathrm{DMI}\,=\,{\textit{b}}_0\,+\,{\textit{b}}_1\textit{ADG}\,+\,{\textit{b}}_2\textit{BW}^{0.75}+\,\varepsilon$, where ${b}_{0}$ is the intercept, ${b}_{1}$ is the linear regression coefficient of the effect of ADG on DMI, ${b}_{2}$ is the linear regression coefficient of the effect of BW^0.75^ on DMI, and $\varepsilon$ is the residual of the equation, i.e., RFI. RWG was obtained as: $\mathrm{ADG}\,={\textit{b}}_0\,+\,{\textit{b}}_1\textit{DMI}\,+{\textit{b}}_2\mathrm{BW}^{0.75}+\,\varepsilon$, where ${b}_{1}$ is the linear regression coefficient for the effect of DMI on ADG, ${b}_{2}$ is the linear regression coefficient for the effect of BW^0.75^ on ADG, and the residual $\varepsilon$ is RWG.

The CG were defined by the test group class (which included sex, year of birth, and breeding program). The test groups consisted of animals that were kept in the same environment and consumed the same diet during the performance test. For data quality control (QC), animals with records for the evaluated traits outside the interval of ± 3.5 standard deviations of CG mean and CG with less than 5 animals were excluded from the analysis. CG size ranged from 22 to 62 animals, with an average of 40.1 ± 11.8 animals. The number of animals and descriptive statistics for each trait after data quality control are shown in Table 1.

**Table 1:** Descriptive statistics of phenotypic information for feed efficiency-related traits in Nellore cattle

Trait^*^	*n*	Mean (± sd)	Min	Max	CG	CV, %
RFI, kg/d	2,321	−0.001 ± 0.65	−3.71	4.84	40	94.68
DMI, kg/d	2,321	7.79 ± 1.66	2.15	13.92	40	21.32
FC, kg DMI/kg gain	2,321	7.52 ± 2.40	3.16	34.82	40	31.99
FE, kg gain/kg DMI	2,321	0.14 ± 0.04	0.03	0.32	40	26.34
RWG, kg/d	2,017	0.001 ± 0.14	−0.82	1.05	40	89.72

*n* Number of observations, *sd* Standard deviation, *Min *and* Max* Minimum and maximum values, *CG* Number of contemporary groups, *CV* Coefficient of variation

^*^RFI Residual feed intake, *DMI* Dry matter intake, *FC* Feed conversion, *FE* Feed efficiency, *RWG* Residual weight gain

### Sequencing data

A total of 171 bulls representing the Brazilian Nellore population were sequenced using either the Illumina HiSeq X™ Ten or the Illumina NovaSeq 6000 platforms. The criteria for selecting the most representative bulls were based on a k-means cluster analysis applied to the genomic relationship matrix of a sample of 100,000 genotyped animals belonging to different Brazilian Nellore breeding programs (DeltaGen, Nelore Qualitas, Cia do Melhoramento, PAINT, and Institute of Animal Science). Within each cluster, the sires with the most genotyped offspring and covering the genotyped population were selected and subsequently sequenced with a genome coverage ranging from 7.81 × to 23.96 ×. Quality control, alignment, and variant calling processes were conducted following the guidelines set by the 1000 Bull Genomes Project. The detailed processes and parameters used for genome sequencing can be found in Fernandes Júnior et al. [18].

A total of 2,744 animals (2,098 with phenotypic information and 646 parents) were genotyped using SNP panels of different densities, including 780 animals with the 770 K high-density panel (Illumina Inc., San Diego, CA, USA), 1,318 with the GeneSeek Genomic Profiler HDi 75 K panel (GeneSeek Inc., San Diego, CA, USA), and 646 with the GeneSeek Genomic Profiler HDi 50 K panel (GeneSeek Inc., San Diego, CA, USA). Markers located in non-autosomal regions or sharing identical genomic coordinates were first removed, and a QC filter excluded autosomal SNPs with a GenCall score < 0.60 to minimize genotyping errors. Subsequently, animals genotyped with medium-density SNP arrays (50 K and 75 K) were imputed to the high-density panel using a reference population of 6,862 animals and FImpute v3 software [19], achieving an imputation accuracy of 0.98.

All 2,744 HD imputed animals were further imputed to WGS level using the 171 sequenced bulls as the reference population. The WGS imputation was performed with FImpute v3 software [19], considering the ARS-UCD1.3 bovine reference map [20], and the imputation accuracy was expected to be greater than 0.94, as previously reported by Fernandes Júnior et al. [18]. After imputation, a total of 29,706,777 single nucleotide polymorphisms (SNPs) in autosomal regions remained. Considering the large number of markers, we performed a linkage disequilibrium (LD) pruning in PLINK 2.0 [21], scanning the genome in windows of 1,000 SNPs, shifting the window by 50 SNPs at a time, and removing one SNP from each pair with *r*^2^ > 0.95 within each window. This procedure reduced redundancy among highly correlated markers and adjusted the dataset to the available computational capacity, resulting in the removal of 26,587,281 markers.

The genomic QC for WGS imputed animals was performed using the QCF90 software [22] to remove genetic markers: a) located at the same genomic position, b) with MAF (Minor allele frequency) < 0.05, c) Call rate < 0.90, d) Hardy–Weinberg equilibrium (HWE) > 0.15 (tested by the maximum deviation of observed heterozygote frequency from expected), and e) monomorphics. In addition, samples with a Call rate < 0.90 and Mendelian conflict were also removed. After filtering, 526,896 and 1,383 SNPs were removed by the MAF and HWE criteria, respectively. After QC of genomic information, a total of 2,744 genotyped animals with 2,591,217 SNP markers remained for GWAS analyses.

### Genome-wide association statistical analysis

The animal model used in GWAS analyses included the CG as fixed effect; the linear and quadratic effects of animals’ age at the beginning of the performance test considerate as covariates; and the genetic additive effect as random. All fixed effects and covariates were formally tested for their contribution to the model, and all were statistically significant (*P* < 0.025). The matrix representation of the model is:

$${\boldsymbol{y}} \, \mathrm{=} \, {\boldsymbol{X}}{\boldsymbol{\beta}}\, \mathrm{+} \, {\boldsymbol{Wa}} \, \mathrm{+} \, {\boldsymbol{e}}$$

where, ${\boldsymbol{y}}$ is an observation vector for each trait; ${\boldsymbol{\beta}}$ is the vector of fixed effects; ${\boldsymbol{a}}$ is the vector of genetic additive effects, assumed as ${\boldsymbol{a}}\sim\mathrm{N(0,} \, {\boldsymbol{H}}{\sigma }_{\mathrm{a}}^{2}\mathrm{)}$, where ***H*** is the matrix that combines the pedigree (***A***) and genomic (***G***) matrices, and ${\sigma }_{\mathrm{a}}^{2}$ is the additive genetic variance; ${\boldsymbol{e}}$ is the vector of residual effects, assumed as ${\boldsymbol{e}}\sim\mathrm{N(0,}\;\boldsymbol{I}{\sigma }_{\mathrm{e}}^{2}\mathrm{)}$, where ***I*** is an identity matrix, and ${\sigma }_{\mathrm{e}}^{2}$ is the residual variance; ***X*** and ***W*** are incidence matrices related to ${\boldsymbol{\beta}}$ and ${\boldsymbol{a}}$, respectively.

The genomic estimated breeding values (GEBVs) for all traits were computed using single-step GBLUP (ssGBLUP) and estimated by BLUPF90 + software [23]. In the ssGBLUP procedure, the inverse of the numerator of kinship matrix is replaced by the inverse of the combined matrix of the genomic-pedigree relationship ***H***^**−1**^ [24]:

$${\boldsymbol{H}}^{-1} \, \text{= }\boldsymbol{ }{\boldsymbol{A}}^{-1} \, \mathrm{+} \left[\begin{array}{cc}{\it0}& {\it0}\\ {\it0}& {\boldsymbol{G}}^{-1}- {\boldsymbol{A}}_{22}^{-1}\end{array}\right]$$

where, ***A***^−1^ is the inverse of the relationship matrix based on the pedigree of all animals ${\boldsymbol{A}}_{22}^{-1}$, is the inverse matrix of the relationship coefficients based on the genotyped animals, and ***G***^−1^ is the inverse of the genomic relationship matrix [25], which is described as:

$${\boldsymbol{G}} = \frac{{\boldsymbol{ZZ}}^{\prime}}{\sum\nolimits_{i =1}^{m} {2P}_{i}(1 - P_{i})}$$

where, ***Z*** = (***M**** – ****P***), in which ***M*** is the SNP incidence matrix, with *m* columns representing the number of markers and *n* lines representing the number of sequenced animals. Each element in ***M*** was set to 0, 1, or 2, for genotypes AA, AB, and BB, respectively. ***P*** is the matrix containing the allele frequencies expressed in 2*p*_*i*_, where *p*_*i*_ is the is the frequency of the second allele at *i*^th^ locus.

The effects of SNP were obtained based on animals’ GEBVs, using the POSTGSF90 software [26]. The equation for computing the effect of SNP can be described as:

$$\widehat{\boldsymbol{u}} \, = \text{ } \lambda {\boldsymbol{Z}}\boldsymbol{^{\prime}}{\boldsymbol{G}}^{-1}{\widehat{\boldsymbol{a}}}$$

where, ***u*** is the vector of each SNP effect; ***â*** is the vector with the GEBVs of genotyped animals, which is represented by a function of the SNP effects (***a*** = ***Zu***); *λ* represents weighting factor given as $\it\mathrm{1/}{\sum }_{i\mathrm{=1}}^{m}{{\mathrm{2}p}}_{i}{\mathrm{(1}-p}_{i}\mathrm{)}$.

The *P*-values for the SNP effects were computed according to Aguilar et al. [27], based on the standardized SNP effects as follows: 

$P-{\mathrm{value}}_j\;=\;\mathit2(\mathit1\;-\;\phi\mathit{\left(\left|\frac{{\widehat{\mathrm u}}_j}{\mathrm{sd}{\mathrm{\left({\widehat u}_{\mathit j}\right)}}_{\it i}}\right|\right)})$, where, ${\it\widehat{\it\mathrm{u}}}_{\text{}j}$ is the estimated effect for the *j*^th^ SNP, and $\it{\mathrm{sd(}}{\widehat{\mathrm{u}}}_{\text{}j}\mathrm{)}$ is the standard deviation of ${\it\widehat{\mathrm{u}}}_{\text{}j}$, and *Φ* is the cumulative density function of the normal distribution.

### Candidate genes and functional enrichment

Manhattan plots containing SNP significance expressed as −log_10_(*P*-value) were constructed using the qqman R package [28], to illustrate the chromosomal regions with significant effect (*P* < 10^–5^) on expressing the interest traits. Candidate genes were annotated considering an upstream and downstream interval of 0.25 Mb from the significant SNP using the Ensembl annotation release 113 [29] considering the ARS-UCD1.3 bovine reference genome. A training list containing genes associated with relevant keywords (feed intake, satiety, digestion, intestine absorption, energy metabolism, energy homeostasis, lipid metabolism, fatty acid metabolism, muscle development, and weight gain) was created using GUILDify web server [30]. The ToppGene Suite [31] was used to prioritize the annotated genes from Ensembl based on genes from training list. The prioritized significant genes were selected based on an overall *P* ≤ 10^−3^, indicating that the test genes have the same functional profile as the genes on the trained list. The prioritized candidate genes list was subjected to functional enrichment analysis via clusterProfiler v4.13.3 package [32] for R, covering terms related to Biological Processes Gene Ontology (GO terms) and Kyoto Encyclopedia of Genes and Genomes (KEGG pathways), considering the *Bos taurus* as background (ARS-UCD1.3 bovine assembly genome).

## Results

### Significant regions and candidate genes

**Fig. 1:**
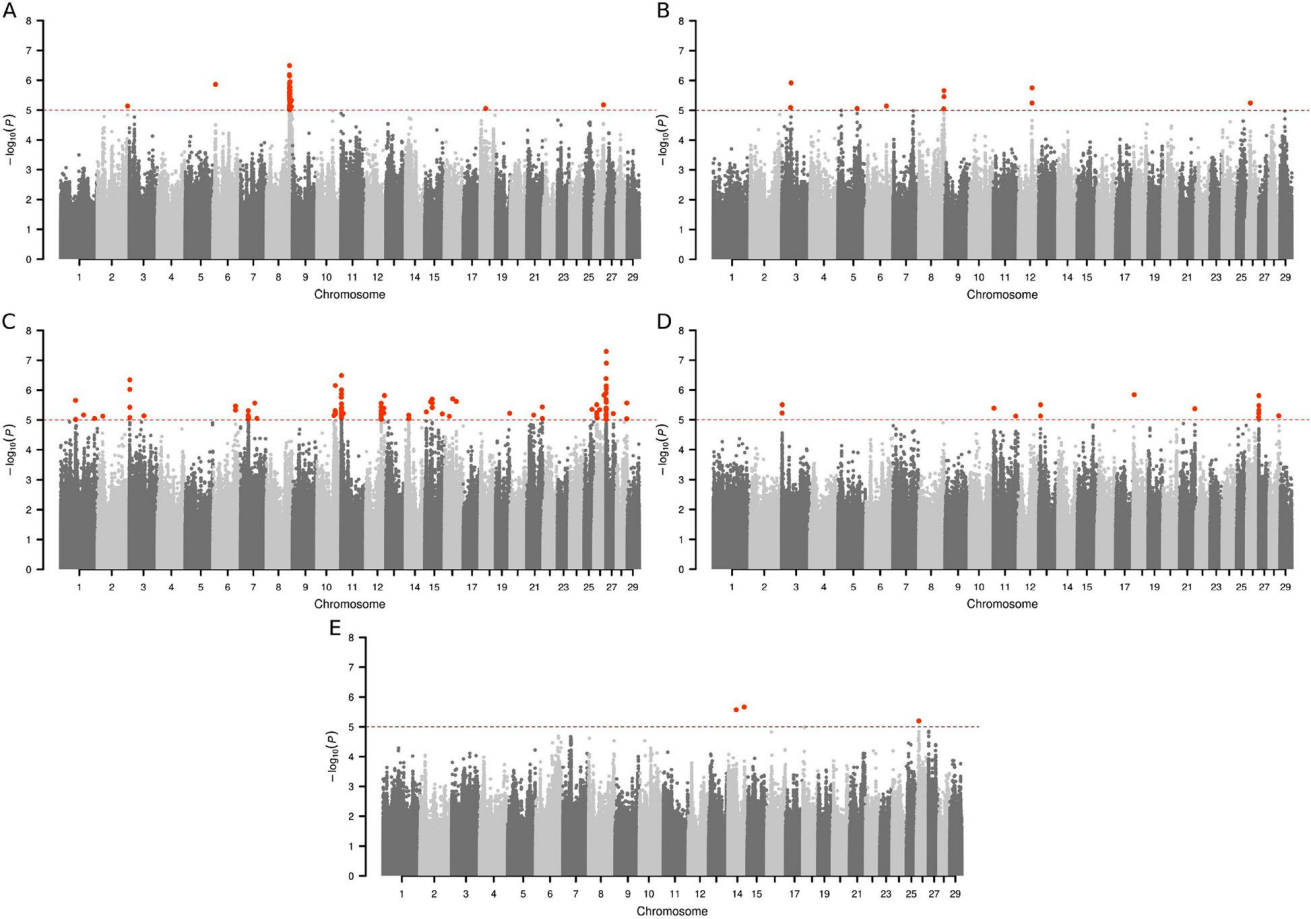
Manhattan plot of genome-wide association analyses showing the significant SNPs (highlighted in orange above the red significance threshold line, −log10 = 5.0) associated with feed efficiency-related traits in Nellore cattle: (**A**) RFI, (**B**) DMI, (**C**) FC, (**D**) FE, and (**E**) RWG

A total of 42 SNPs exceeded the threshold (*P* < 10^–5^) for RFI, distributed on *Bos taurus* autosomes (BTA) 2, 6, 8, 18, and 26 (Fig. 1A). A total of 62 candidate genes were annotated within the SNP regions (± 0.25 Mb of SNP) for RFI (Table S1). For DMI, 10 SNPs located on BTA 3, 5, 6, 8, 12, and 26 were found (Fig. 1B), in regions harboring 15 positional genes (Table S2). A total of 99 markers were significantly associated with FC in GWAS analysis. These significant SNPs were found in chromosomal regions on BTA 1, 2, 3, 6, 7, 10, 11, 12, 14, 15, 16, 19, 21, 25, 26, 27, and 28 (Fig. 1C), harboring 201 candidate genes (Table S3). For FE, 15 SNPs distributed across BTA 3, 11, 13, 18, 21, 27, and 28 (Fig. 1D), close or within 53 positional genes (Table S4) were found. For RWG, two SNPs were found on BTA 14 and one SNP on BTA 26 (Fig. 1E), on genomic regions with 11 candidate genes (Table S5). Inspection of the Quantile–Quantile (Q-Q) plots did not show any potential inflation or deflation in the results of genome-wide association studies when using imputed whole-sequencing data (λ ≈ 1). All Q-Q plots are presented in Fig. S1. Additionally, four genomic regions with overlapping SNP windows within ± 0.25 Mb were identified, revealing shared QTL across traits. For RFI and DMI, a single overlapping region was detected on BTA8 (107.86–108.38 Mb). For FC and FE, three additional shared regions were identified: one on BTA11 (1.59–2.88 Mb) and two on BTA27 (0–0.65 Mb and 0.70–1.70 Mb). These findings indicate that FC and FE share multiple QTL with potential pleiotropic effects, which is expected given that both traits describe the animal’s ability to convert feed intake into productive output and are therefore closely related indicators of feed efficiency. After gene prioritization by ToppGene, a total of 21, 9, 68, 23, and 8 positional candidate genes were retained for RFI, DMI, FC, FE, and RWG, respectively. The prioritized candidate genes for each trait are presented in Tables 2, 3, 4, 5, 6.

**Table 2:** Chromosome (Chr), position, gene identification, and overall *P*-value of ToppGene prioritization for RFI

Chr	Position, bp	Gene	Overall *P*-value
2	129,321,545	*E2F2*	9.77e-04
2	129,477,138	*ZNF436*	7.78e-03
2	129,769,257	*KDM1A*	5.69e-05
6	10,520,964	*NDST4*	4.05e-03
6	10,929,100	*UGT8*	1.37e-03
8	99,799,187	*TXN*	2.05e-03
8	99,897,703	*SVEP1*	9.19e-03
8	100,188,491	*MUSK*	8.20e-04
8	100,355,969	*LPAR1*	9.84e-04
8	100,963,670	*PTGR1*	2.14e-03
8	101,283,115	*UGCG*	1.25e-03
8	102,470,339	*SLC31A1*	1.65e-03
8	105,351,875	*PAPPA*	5.44e-03
18	23,753,085	*MMP2*	8.65e-07
18	23,786,253	*LPCAT2*	9.36e-05
18	23,885,619	*SLC6A2*	2.98e-04
18	23,948,830	*CES1*	1.28e-04
18	24,052,455	*GNAO1*	6.70e-04
18	24,056,405	*BBS2*	1.90e-06
18	24,207,326	*AMFR*	1.77e-03
26	41,450,677	*FGFR2*	3.77e-05

**Table 3:** Chromosome (Chr), position, gene identification, and overall *P*-value of ToppGene prioritization for DMI

Chr	Position, bp	Gene	Overall *P*-value
3	40,305,057	*COL11A1*	1.85e-03
5	82,983,629	*ITPR2*	3.79e-05
6	89,093,080	*MTHFD2L*	9.54e-04
6	89,244,813	*EPGN*	2.12e-03
6	89,306,919	*EREG*	7.33e-04
6	89,379,645	*AREG*	6.92e-05
8	107,057,826	*TLR4*	4.66e-05
26	16,743,890	*PDLIM1*	4.08e-03
26	16,822,448	*SORBS1*	5.56e-04

**Table 4:** Chromosome (Chr), position, gene identification, and overall *P*-value of ToppGene prioritization for FC

Chr	Position, bp	Gene	Overall *P*-value
1	64,646,714	*GSK3B*	2.78e-04
1	145,848,081	*FTCD*	4.31e-04
1	145,889,102	*LSS*	1.58e-03
1	146,001,687	*PCNT*	2.09e-03
2	22,797,247	*SP3*	1.43e-03
3	3,105,779	*TMCO1*	8.52e-03
7	61,380,054	*CSF1R*	4.76e-04
7	61,474,756	*PDGFRB*	5.18e-05
7	61,521,982	*CDX1*	5.70e-03
7	61,576,011	*CAMK2A*	4.79e-04
7	61,747,923	*CD74*	2.13e-03
7	61,815,017	*NDST1*	1.47e-03
7	70,896,232	*IL12B*	9.27e-04
10	73,436,691	*PRKCH*	3.02e-03
10	78,562,589	*GPHN*	1.03e-03
10	79,462,217	*PIGH*	9.38e-04
10	79,499,357	*ARG2*	5.90e-03
10	79,555,431	*RDH11*	8.99e-04
10	79,580,110	*RDH12*	1.38e-04
10	79,599,601	*ZFYVE26*	4.46e-03
11	1,585,412	*BUB1*	1.24e-03
11	1,697,163	*NPHP1*	2.06e-04
11	2,017,963	*PROM2*	1.63e-03
11	2,042,314	*KCNIP3*	4.11e-03
11	2,318,512	*STARD7*	3.06e-03
11	2,557,667	*ARID5A*	2.12e-03
11	2,800,346	*ANKRD23*	6.63e-03
11	2,947,386	*COX5B*	5.10e-04
11	3,047,488	*ZAP70*	4.98e-03
11	10,475,416	*MOB1A*	6.08e-03
11	10,696,134	*DGUOK*	4.33e-04
11	10,737,085	*ACTG2*	1.67e-03
11	10,889,044	*ALMS1*	3.76e-05
14	14,779,050	*TRIB1*	3.78e-03
14	14,849,669	*NSMCE2*	4.83e-03
15	7,313,317	*TRPC6*	3.35e-03
15	75,940,787	*PEX16*	2.35e-03
15	76,283,233	*CREB3L1*	6.43e-04
15	76,330,895	*DGKZ*	1.63e-03
15	76,374,876	*MDK*	1.72e-03
15	76,389,709	*AMBRA1*	2.66e-03
16	35,395,729	*RGS7*	8.05e-03
16	51,197,668	*CPTP*	3.09e-03
16	51,238,668	*UBE2J2*	4.06e-03
16	51,422,882	*AGRN*	3.85e-03
16	51,514,432	*NOC2L*	1.35e-03
16	51,602,848	*ZBTB17*	8.00e-03
16	51,633,019	*SPEN*	7.49e-03
19	61,089,024	*MAP2K6*	1.85e-03
25	34,159,242	*POR*	3.54e-05
25	34,254,236	*MDH2*	2.13e-05
25	34,345,264	*HSPB1*	1.84e-05
25	34,370,215	*YWHAG*	7.07e-03
25	34,676,914	*SH2B2*	3.95e-03
26	12,372,324	*HTR7*	4.11e-03
26	12,533,108	*ANKRD1*	8.08e-05
26	14,403,229	*CYP26C1*	1.41e-03
26	14,416,061	*CYP26A1*	7.07e-05
26	14,623,106	*MYOF*	3.32e-04
26	44,045,036	*OAT*	4.49e-03
27	1,239,143	*CLN8*	9.73e-04
27	1,452,630	*MYOM2*	1.67e-04
28	43,578,135	*DRGX*	5.28e-03
28	43,632,381	*ERCC6*	1.27e-04
28	43,744,775	*CHAT*	3.79e-03
28	43,900,420	*PARG*	3.50e-03
28	44,036,736	*NCOA4*	4.53e-03
28	44,228,060	*MARCHF8*	3.49e-03

**Table 5:** Chromosome (Chr), position, gene identification, and overall *P*-value of ToppGene prioritization for FE

Chr	Position, bp	Gene	Overall *P*-value
3	1,259,432	*CD247*	9.52e-04
3	1,280,068	*POU2F1*	3.26e-04
3	1,903,517	*ILDR2*	2.89e-03
11	2,017,963	*PROM2*	2.53e-03
11	2,042,314	*KCNIP3*	2.63e-03
11	2,164,621	*GPAT2*	5.49e-04
11	2,251,377	*ADRA2B*	4.77e-03
11	2,318,512	*STARD7*	5.10e-03
11	2,355,706	*TMEM127*	4.31e-03
11	95,924,970	*SCAI*	7.55e-03
11	96,115,159	*HSPA5*	4.13e-04
11	96,142,919	*GAPVD1*	3.27e-03
11	96,283,685	*MAPKAP1*	1.99e-03
18	5,240,541	*WWOX*	1.90e-05
21	65,168,061	*YY1*	2.91e-05
21	65,203,062	*SLC25A29*	1.26e-03
21	65,238,072	*WARS1*	6.28e-03
21	65,625,842	*DLK1*	8.83e-05
27	1,239,143	*CLN8*	1.93e-03
27	1,452,630	*MYOM2*	1.29e-03
28	41,308,582	*OPN4*	1.63e-03
28	41,325,440	*LDB3*	1.71e-04
28	41,486,119	*BMPR1A*	6.25e-04

**Table 6:** Chromosome (Chr), position, gene identification, and overall *P*-value of ToppGene prioritization for RWG

Chr	Posiiton, bp	Gene	Overall *P*-value
14	35,611,151	*TRPA1*	2.82e-03
14	35,874,421	*KCNB2*	9.34e-03
14	70,467,827	*TMEM67*	4.43e-04
14	70,563,223	*CIBAR1*	7.77e-03
26	10,626,814	*ACTA2*	4.12e-04
26	10,678,448	*FAS*	1.02e-05
26	10,940,264	*CH25H*	4.17e-04
26	10,952,398	*LIPA*	8.86e-06

### Functional enrichment

**Fig. 2:**
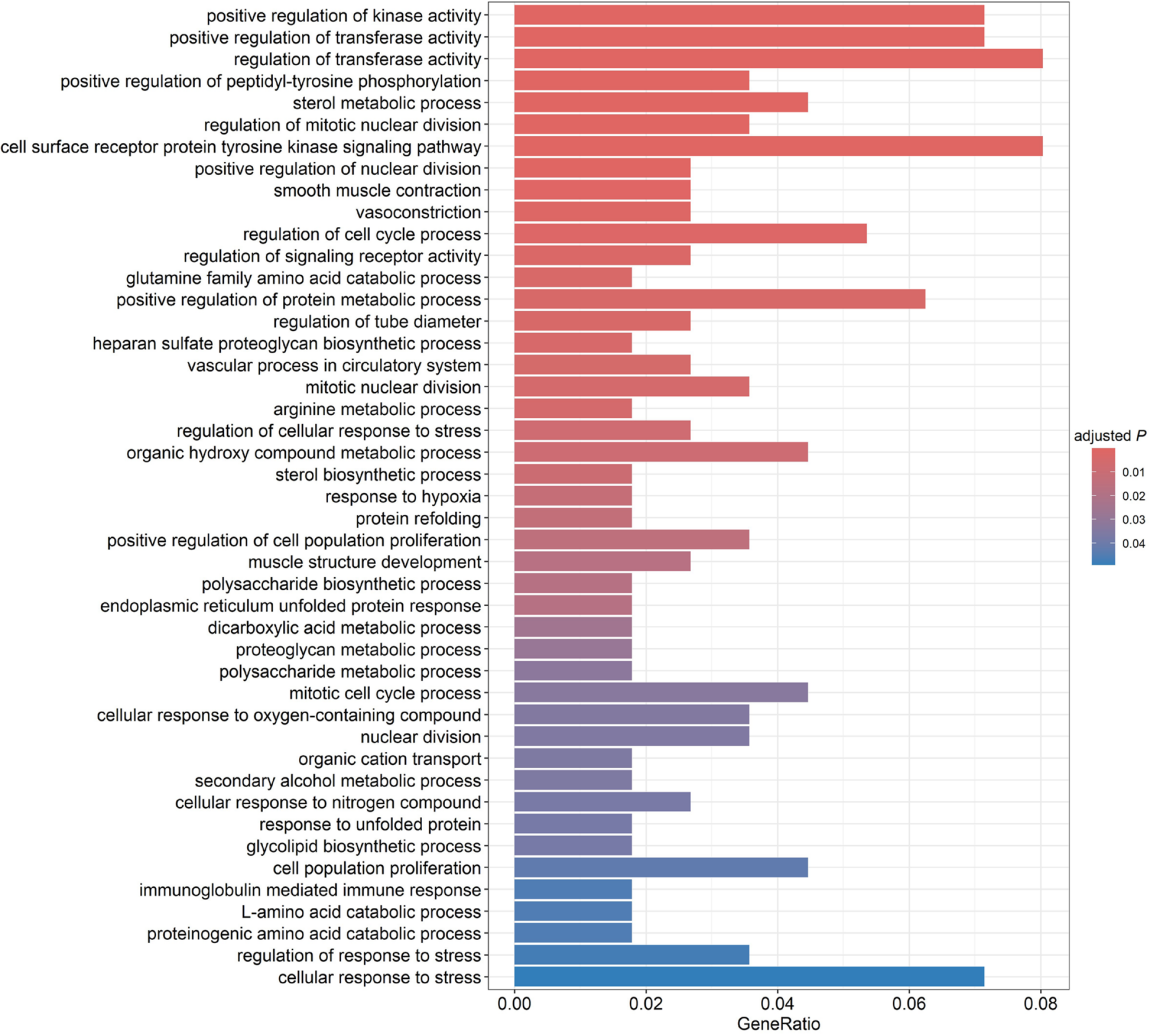
Barplot Biological Process GO enrichment analysis of prioritized genes. The *x* axis represents the number of genes, *y* axis the significant enriched GO term, and bar colors represents adjusted values with blue being the highest and red the lowest adjusted *P*-values

In the functional analysis, the relationships between prioritized genes and Biological Processes GO terms enriched for the studied traits were identified (Fig. 2). The complete table containing all GO terms enrichment results can be found in Table S6. Among the main processes identified, those related to nutrient metabolism, cell cycle regulation, stress response, and cell proliferation stand out. The metabolic processes included pathways such as "sterol metabolic process", "arginine metabolic process" and "glutamine family amino acid catabolic process", indicating the central role of lipid and amino acid metabolism in feed efficiency. In addition, polysaccharide and proteoglycan biosynthesis processes, such as "polysaccharide biosynthetic process" and "heparan sulfate proteoglycan biosynthetic process", were identified as significant processes. Cell cycle regulation pathways, including "mitotic nuclear division", "nuclear division", and "positive regulation of cell population proliferation", suggest a strong association between growth/cell proliferation and feed efficiency. These processes were accompanied by stress response mechanisms, such as "response to hypoxia" and "regulation of cellular response to stress", which reflect the adaptive capacity of animals under different environmental and nutritional conditions. Additionally, organic compound transport and metabolism processes, such as "organic cation transport" and "secondary alcohol metabolic process", reinforce the important role of efficient nutrient transport in feed efficiency.

**Fig. 3:**
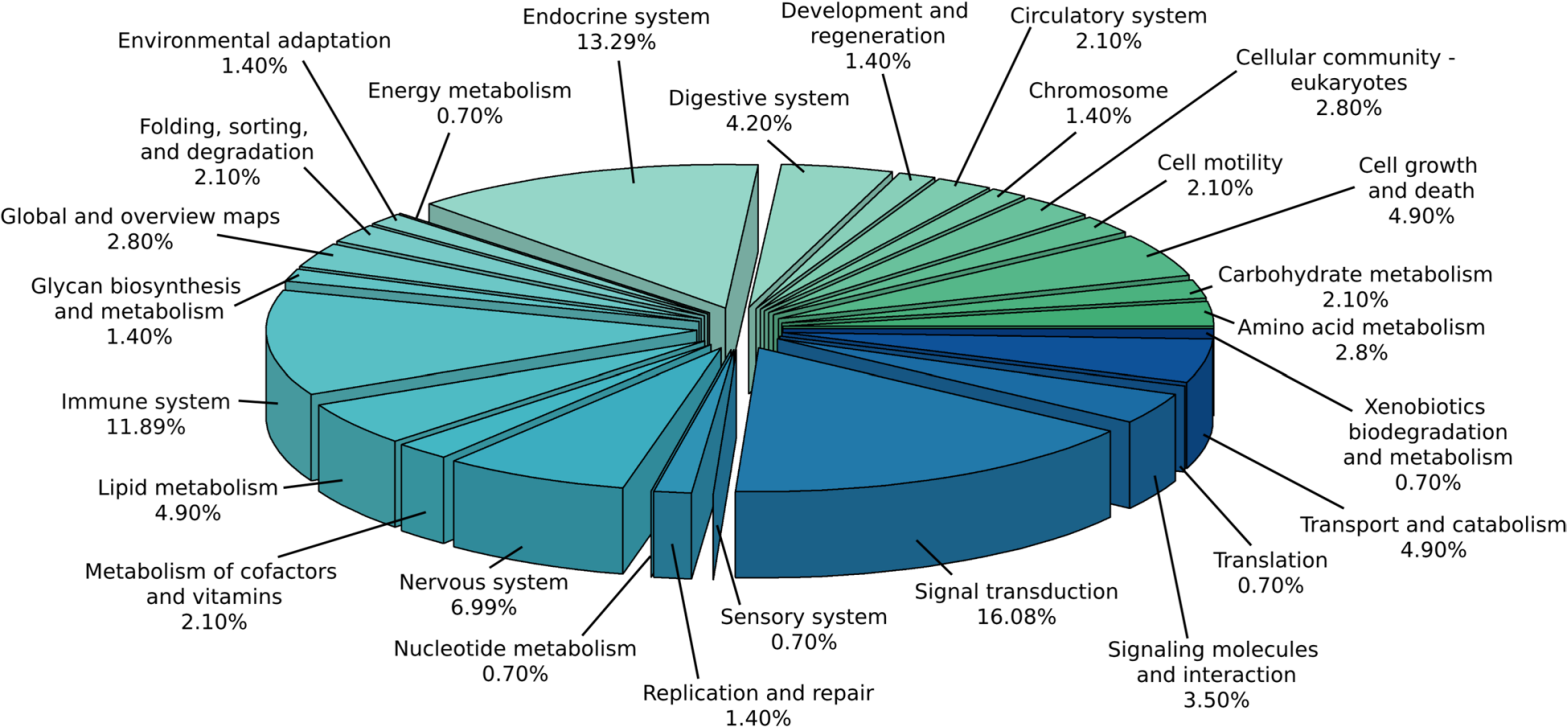
KEGG pathway categories enriched for prioritized genes associated with feed efficiency-related traits in Nellore cattle

The KEGG pathways enriched for the studied traits in the functional analysis are presented in Fig. 3 and Table S7. Signal transduction was the most enriched category, representing 16.08% (23 KEGG entries, such as MAPK, PI3K-Akt, mTOR, Rap1, calcium, cAMP, cGMP-PKG and TGF-β signaling pathways) of the total identified pathways. Next, the endocrine system showed high representation, with 13.29% (19 KEGG pathways, including insulin, glucagon, growth hormone synthesis/secretion, thyroid hormone, estrogen, among other signaling pathways), followed by the immune system representing 11.89% (17 KEGG entries) of total. The Nervous system contributed with 6.99% represented by 10 KEGG pathways (e.g, cholinergic, dopaminergic, serotonergic, GABAergic, and glutamatergic synapses), while pathways associated with cell growth and death, lipid metabolism, and transport and catabolism presented a contribution of 4.9% (7 pathways) each. Additionally, the digestive system represented 4.2% of the total, with 6 KEGG entries, and pathways related to signaling molecules and interaction contributed with 3.5% through 5 KEGG entries. Metabolic pathways, such as amino acid metabolism (including arginine, histidine, cysteine, and methionine metabolism) and categories linked to cellular community—eukaryotes, were identified representing 2.8%. Other categories, such as carbohydrate metabolism (e.g, citrate cycle – TCA cycle and pyruvate metabolism signaling pathways), cell motility, and circulatory system, showed low representation, with 2.1% (3 KEGG pathways each). Finally, less representative categories, including nucleotide metabolism, translation, and energy metabolism (oxidative phosphorylation) represented 0.7% (1 KEGG pathway each) of the total.

## Discussion

### Candidate genes associated with feed efficiency-related traits

#### Residual feed intake

Three muscle-related genes (*FGFR2*, *MUSK*, and *PAPPA*; Table 2) were found to be associated with RFI. Feed efficiency is directly associated with the body's ability to convert nutrients into functional tissues and muscle. *FGFR2* plays a role in satellite cells’ proliferation and development during skeletal muscle myogenesis [33]. In an association study, Delpuech et al. [34] highlighted *FGFR2* as a potential candidate gene within a QTL for RFI in pigs. Additionally, this gene was upregulated in high-feed efficient broilers [35]. *MUSK* is essential for formation and maintenance of neuromuscular junctions and is critical for maintaining muscle mass [36]. Previously, *MUSK* was found in a candidate region for body composition and structural soundness traits in pigs [37]. The *PAPPA* gene encodes an enzyme responsible for mediating the hydrolysis of insulin-like growth factor (IGF) binding proteins, thus increasing IGF bioavailability and inducing IGF signaling [38]. Recent researches have linked the *PAPPA* gene to traits such as growth, carcass, and meat quality in pigs [39] and chickens [40].

Other possible candidate associated with RFI is *BBS2* (Table 2), a gene involved in obesity, lipid metabolism, and immune response. Mice with *BBS2* knockout exhibit a deficiency in transporting leptin receptors to the cell membrane, leading to obesity due to the development of leptin resistance [41]. Leptin is a hormone produced primarily by adipose tissue that is intrinsically involved in modulating feed efficiency. It acts through central neural pathways to regulate feed intake [42]. Additionally, leptin regulates energy balance and body weight by suppressing appetite and increasing energy expenditure [43].

The *UGCG* gene (Table 2) encodes glucosylceramide synthase, a key enzyme in the glycosphingolipid (GSL) biosynthesis pathway. GSLs are integral components of cell membranes and are involved in regulating metabolic pathways that influence growth and energy utilization [44]. Furthermore, it has been observed that GSLs are essential for the endocytic function of intestinal cells, facilitating the absorption of dietary lipids and other nutrients [45].

*TXN* (Table 2) is a regulator of cellular responses to oxidative stress. Oxidative stress is a biochemical phenomenon that negatively affects growth, nutrient absorption, and feed utilization efficiency, thus compromising animal performance [46]. *TXN* overexpression can improve glucose tolerance and reduce insulin resistance, contributing to increased availability of energy for growth [47]. In chickens, genes encoding antioxidant proteins, including *TXN*, have been reported to be upregulated in high-feed efficiency animals [48].

#### Dry matter intake

The *EPGN*, *EREG*, and *AREG* genes (Table 3) encode proteins that are part of the epidermal growth factor (EGF) family. EGF signaling triggers a cascade of other intracellular signaling pathways, including the MAPK and PI3K/Akt pathways, crucial for cellular responses that influence growth and metabolism [49]. In the gastrointestinal tract (GIT), EGF acts as a mucosal protective factor, influencing intestinal development and maintaining epithelial cell homeostasis [50]. Studies have shown that orally administered EGF improved intestinal development and health [51], nutrient digestibility, and increased mRNA expression of nutrient transporters [52].

The *ITPR2* (Table 3) is involved in nutrient metabolism, acting as a calcium (Ca^2+^) channel of pancreatic acinar cells, mediating the regulation of pancreatic exocrine secretion [53]. It has been reported that *ITPR2* knockout can result in exocrine dysfunction, compromising nutrient digestion and causing reduced body weight in mice [54]. Also, *ITPR2* regulates Ca^2+^ fluxes from the endoplasmic reticulum to mitochondria, contributing to energy production through oxidative phosphorylation, a calcium-dependent process [55]. *TLR4* is involved in intestinal microbiota regulation, where its overexpression can improve microbial diversity and favor bacteria producing short-chain fatty acids [56]. Additionally, *TLR4* signaling in dopaminergic neurons impacts feeding behavior by influencing motivation and feed preferences [57]. Investigating transcriptome data, Yang et al. [58] highlighted *TLR4* as a key gene affecting feed efficiency in chickens.

#### Feed conversion

Multiple genes related to skeletal muscle metabolism were found in association with FC (Table 4), establishing a relationship between feed efficiency and growth. *AGRN* encodes the agrin, a protein secreted by motor neurons and is involved in the *MUSK* phosphorylation [59], a gene associated with RFI in this study and is also involved in muscle mass [36]. The *MYOM2* and *MYOF* genes encode essential structural proteins in skeletal muscle, playing crucial roles in maintaining the integrity and functionality of muscle fibers [60, 61]. *ANKRD1* and *ANKRD23* belong to the muscle ankyrin repeat protein family, involved in skeletal muscle structure and function. *ANKRD1* participates in biological processes related to myogenesis, such as skeletal muscle tissue development and muscle cell differentiation [62]. *ANKRD23* is involved in myoblast differentiation, specialized cells that give rise to muscle tissue [63]. Both *ANKRD1* and *ANKRD23* genes are associated with feed conversion rate in broilers [64] and pigs [65]. *HSPB1* plays an essential role in myofibrillar structure organization and muscle mass regulation [66]. Carvalho et al. [67] observed a greater *HSPB1* abundance in the skeletal muscle of more feed-efficient steers compared to less efficient. This increase may be associated with a lower protein turnover rate and, consequently, lower energy expenditure in skeletal muscle, indicating that this metabolic difference plays a direct role in the superiority of more efficient animals [67].

The *ARG2* gene (Table 4) encodes arginase, a protein that participates in excess nitrogen elimination from amino acid metabolism [68]. In pigs, gene expression patterns combined with functional annotation identified *ARG2* as a candidate biomarker related to feed efficiency [69]. In Nellore cattle, *ARG2* was associated with four feed efficiency phenotypes, including feed conversion [70]. *COX5B* is a structural component of the cytochrome C oxidase (COX) complex, a protein complex that plays a terminal role in the mitochondrial respiratory chain. COX is responsible for oxygen consumption in mitochondrial oxidative phosphorylation, determining the rate of ATP synthesis in cells [71] and therefore playing a key role in energy metabolism regulation.

Two genes (*HTR7* and *CHAT*; Table 4) related to neurotransmission were identified within significant regions associated with feed conversion. The *HTR7* gene encodes a serotonin receptor that is involved in food intake and body weight regulation. This gene is expressed in GIT smooth muscle, enteric neurons, and enterocytes, and has been correlated with intestinal crypt depth in pigs [72], suggesting its involvement in maintenance of proper nutrient absorption function. The *CHAT* gene encodes choline acetyltransferase, an enzyme that plays a significant role in acetylcholine (ACh) synthesis [73]. ACh is a neurotransmitter that regulates gastrointestinal motility [74]. Furthermore, ACh regulates epithelial ion transport and water fluxes in intestinal mammal cells, ensuring necessary intestinal hydration for digestion, nutrient absorption, and lubrication for intestinal content passage [75].

#### Feed efficiency

*POU2F1* and *GPAT2* (Table 5) are two genes involved in growth and lipid metabolism. *POU2F1* belongs to a transcription factors’ family that regulate the activity of multiple genes involved in processes such as cell growth, proliferation, and differentiation [76]. In pigs, this gene has been identified as one of the key transcription factors regulating genes involved in growth and fat deposition [77]. In Nellore cattle, a QTL containing the *POU2F1* gene was associated with visual score traits, explaining 41% of the genetic variance in conformation and 29% in musculature scores [76]. *GPAT2* encodes a mitochondrial enzyme that plays a role in triacylglycerol biosynthesis, the primary energy storage form in mammalian cells [78]. *GPAT2* was associated with growth-related traits as well as fatty acid composition of intramuscular and subcutaneous fat in pigs [79]. In Nellore cattle, this gene was found in association with the *Longissimus* muscle area [80], highlighting its role in growth control.

The *WWOX* gene (Table 5) influences glucose homeostasis, lipid metabolism, and the degradation of proteins and carbohydrates [81]. *WWOX* absence in skeletal muscles reduces mitochondrial oxidation, increases glycolysis in cells and fatty acid oxidation [82], compromising the body's ability to maintain energy homeostasis. *BMPR1A* (Table 5) promotes adipogenesis [83] and is associated with body size in sheep [84]. In mice, *BMPR1A* has been observed to regulate the development of hypothalamic circuits that are critical for feeding behavior [85]. In turn, *HSPA5* interacts with multiple proteins in the endoplasmic reticulum, potentially contributing to protein transport across the cell [86]. In Angus steers subjected to feed restriction, *HSPA5* exhibited upregulated expression, indicating its potential involvement in adaptive mechanisms that optimize feed utilization [87]. Moreover, studies have shown that more feed-efficient cattle have greater hepatic *HSPA5* expression [86, 88].

The identified genes suggest that FE arises from a balance of growth, energy metabolism, tissue deposition, and stress responses, linking metabolic efficiency to animals with greater growth potential and improved body composition. These animals exhibit better metabolic adaptation, utilizing energy effectively while maintaining homeostasis under conditions like food restriction. These findings emphasize that feed efficiency depends not just on food intake, but on the organism's ability to convert it effectively into energy and tissues, minimizing losses and optimizing performance.

#### Residual weight gain

The *FAS* gene (Table 6) is involved in hepatic lipid metabolism regulation, mitochondrial function, and fatty acid oxidation [89]. Connor et al. [87] observed that increased mitochondrial function is associated with improved feed efficiency in cattle. *FAS* expression was also correlated with body mass index, adipocyte macrophage infiltration, and insulin resistance [90]. In Nellore cattle, a transcriptome study identified *B2M*, a biomarker with regulatory control over *FAS*, as being overexpressed in the liver of low-RFI animals [91]. These reports highlight the possible involvement of *FAS* in the regulation of feed efficiency.

The *TRPA1* (Table 6) encodes an integral membrane protein responsible for cation-selective channels’ formation. Rosendahl et al. [92] observed *TRPA1* expressed at mRNA level in the ruminal epithelium of cattle and sheep. The authors provided functional evidence that *TPRA1* acts as an uptake channel for Na^+^, Ca^2+^, and ammonia in NH_4_^+^ form in the rumen [92]. Ruminal microorganisms degrade dietary proteins and nitrogenous compounds into peptides, amino acids, and, eventually, ammonia (both in NH_3_ and NH_4_^+^ forms). However, excessive accumulation of ammonia compromises the animal's ability to utilize nitrogen efficiently. In this way, excess ammonia is absorbed by the ruminal epithelium and transported to the liver, where it is metabolized into urea. Ultimately, urea can be excreted via urine or recycled into the rumen, which can serve as a substrate for microbial protein synthesis, an excellent protein source for muscle synthesis [93]. In this context, *TRPA1* may be involved in urea recycling, acting as a channel for NH_4_^+^ translocation to the liver, reflecting better feed use to produce proteins destined for muscle growth. In agreement, Wu et al. [94] identified polymorphisms in the *TRPA1* gene, which were significantly associated with several growth traits in cattle.

*LIPA* and *CH25H* (Table 6) are genes that play key roles in cholesterol metabolism. *LIPA* is responsible for hydrolyzing cholesteryl esters and triglycerides within lysosomes, while *CH25H* inhibits cholesterol biosynthesis [95]. Cholesterol is involved in several metabolic processes, including the synthesis of steroid hormones and bile acids, which are essential for fat digestion and absorption [96]. Additionally, cholesterol can serve as a biomarker for evaluating feed efficiency. For example, increased feed intake exceeding metabolic needs was linked to elevated plasma cholesterol levels in pigs [97]. This is reinforced by Bourgon et al. [98], who observed lower serum cholesterol levels in more efficient cattle than in the inefficient ones.

The identified genes, which are linked to lipid metabolism, cholesterol regulation, and nitrogen utilization, influence RWG. The increased expression of *FAS* in more efficient animals suggests a greater capacity for energy regulation in the liver. *TRPA1* may enhance urea recycling and microbial protein synthesis, optimizing nitrogen compound utilization. *LIPA* and *CH25H* contribute to cholesterol management, indicating a more efficient metabolism. These mechanisms imply that more efficient animals utilize nutrients more effectively, promoting growth while minimizing energy waste.

### Biological process GO terms for prioritized candidates

#### Nutrient metabolism and biosynthesis

Steroid (GO:0016126, GO:0016125; Table S6) and amino acid metabolism (GO:0170035, GO:0170040; Table S6) are directly linked to energy production and protein synthesis [99], which are essential for muscle growth and body development. For example, testosterone, an essential steroid hormone, is particularly important in promoting muscle mass and strength, and may influence glucose and amino acid metabolism, as well as mitochondrial metabolism [100]. A study in beef cattle treated with androgen and estrogen-based compounds reported beneficial and additive effects, resulting in significant improvements in growth and feed efficiency [101].

Furthermore, processes related to proteoglycans (GO:0015012, GO:0006029; Table S6) and polysaccharide biosynthesis (GO:0005976, GO:0000271; Table S6) contribute to tissue growth and maintenance, optimizing the use of ingested nutrients. In the extracellular matrix, proteoglycans are known to stabilize and protect growth factors from proteolysis, ensuring their availability for cell signaling [102]. Additionally, genes involved in proteoglycan signaling have previously been associated with RFI and DMI in Nellore cattle [70].

#### Stress responses and metabolic adaptation

Oxidative stress is an inevitable consequence of aerobic metabolism since mitochondrial ATP production is a process that generates reactive oxygen species (ROS) in mammals [103]. Oxidative stress occurs when ROS and other free radicals exceed an organism's antioxidant capacity, resulting in increased energy expenditure to neutralize these oxidizing agents [104]. The endoplasmic reticulum (ER) is highly sensitive to physiological changes, such as variations in redox state caused by ROS accumulation, nutrients and Ca^2+^ levels, protein synthesis rate, and responses to pathogens and inflammation [105]. These changes can lead to the accumulation of unfolded or misfolded proteins, triggering ER stress. Thus, the endoplasmic reticulum unfolded protein response (GO:0030968, GO:0006986; Table S6) has the role of mitigating the damage associated with ER stress [106]. In a bovine liver proteome study, Fonseca et al. [107] observed an abundance of proteins involved in the prevention and correction of protein assembly and folding disorders in high-efficiency animals, suggesting that these animals have a greater capacity to adapt to stress, demanding lower energy expenditure. Similarly, Casal et al. [103] observed a higher hepatic antioxidant capacity in steers with low RFI, indicating that reduced hepatic oxidative stress should reduce maintenance requirements and improve energy use efficiency.

#### Organic compounds' transport and use

Organic substances regulate processes that alter cellular state or activity in response to a specific substance [108]. In this context, we could suppose that the cellular response to these substances should lead to metabolic pathway signaling involved in feed efficiency modulation. Studies conducted in broilers observed positive effects of organic compounds on growth and feed efficiency, intestinal morphology, and nutrient utilization [109, 110]. In addition, short-chain fatty acids (such as acetic, propionic, and butyric acids) are organic compounds produced by enteric fermentation and serve as a primary energy source for ruminants. These organic acids also ensure ruminal microbiome health, maintaining its proper function in providing nutrients and energy for animal production [111]. Thus, efficient metabolism and transport of critical metabolites ensure that the organism has rapid access to energy and nutrient sources, optimizing animal growth and efficiency.

### KEGG pathways' enrichment

#### Signal transduction

The MAPK signaling pathway (bta04010) is well documented in the literature for its role in regulating feed efficiency. Several research have shown that MAPK signaling regulates energy balance [112] and growth and development [113]. Different studies conducted in cattle have found genes involved in the MAPK signaling pathway associated with feed efficiency phenotypes [114, 115], including in Nellore cattle [17]. The PI3K-Akt pathway (bta04151) is involved in glucose, lipid, and energy metabolism, and was previously enriched for genes obtained by duodenal RNA-Seq from Chinese beef cattle divergent in feed efficiency [115]. mTOR signaling (bta04150) in the hypothalamus increases the expression of neuropeptides that promote feeding behavior [116] and its dysfunction is implicated in the development of hyperphagia, weight gain, leptin resistance, and obesity [117]. Moreover, mTOR is crucial for regulating protein synthesis and nutrient utilization [118]. Rap1 (bta04015) has diverse roles in several neuronal functions that regulate energy balance, glucose homeostasis, and leptin actions [119], which are mechanisms fundamental to optimizing feed efficiency. This evidence is supported by Oliveira et al. [120], who found enriched target genes for the Rap1 pathway expressed in the skeletal muscle of more feed-efficient cattle.

Ca^2+^ signaling (bta04020) also plays a key role in regulating feed efficiency. It is known that intracellular calcium levels can influence several metabolic processes, including those related to lipid metabolism and energy expenditure, in addition to regulating food intake through neuronal excitability regulation [121]. cAMP (bta04024) and cGMP (bta04022) are ubiquitous types of second messengers involved in diverse biological functions. Ca^2+^ and cAMP signaling pathways regulate lipid droplet lipolysis in adipocytes [122], which is crucial for energy mobilization. In addition, hypothalamic cAMP abundance appears to stimulate feed intake [123]. Ca^2+^, cAMP, and cGMP-PKG pathways have previously been associated with feed efficiency traits in cattle of different breeds [15, 17].

Transforming growth factor beta (TGF-β) signaling (bta04350) is involved in cell growth, differentiation, and apoptosis, and in the regulation of muscle tissue development [124]. Oliveira et al. [120] point to skeletal muscle growth as an indicator of feed efficiency. This hypothesis is supported by studies that observed that more efficient animals have a larger *Longissimus* muscle area [125]. However, protein deposition is a process that demands a higher energy cost compared to fat deposition, partly due to protein turnover [126]. Therefore, based on these information, we infer that animals with greater feed efficiency are energetically more efficient in protein deposition for muscle synthesis, presenting a lower protein turnover rate and, thus, better using the protein and metabolizable energy from consumed feed.

#### Nervous system

“Ergic” synapses occur on neurons that utilize specific neurotransmitters and have been implicated in signal transmission to regulate diverse peripheral functions such as feed intake control and gastrointestinal functions. For example, the dopaminergic (bta04728) system regulates motivational behavior, including feeding behaviors such as appetite control and motivational and emotional drives linked to intake [127]. In mice, dopamine receptors’ depletion led to reduced food intake and body weight, concomitantly with increased basal energy expenditure and leptin sensitivity [128]. The serotonergic (bta04726) system is implicated in regulating feed intake by amplifying and prolonging satiety signals [129]. A study on dietary tryptophan supplementation, a serotonin precursor, showed increased hypothalamic serotonin levels in pigs alongside improved feed efficiency [130]. The GABAergic (bta04727) system appears to play a role in feeding behavior by stimulating hyperphagia. In lactating Holstein cows supplemented with GABA, a linear increase in DMI and milk production was observed concurrently with increasing GABA levels [131]. Glutamate (bta04724) is essential for regulating intestinal secretion and motility through the brain-gut axis [132]. It also modulates neuronal pathways that control critical digestive functions, including taste perception and nutrient absorption [133]. Studies have identified key genes related to glutamatergic signaling that influence FC in pigs [134] and RFI in beef cattle [135].

#### Endocrine system

GIT is the first system that detects the diet and the energy present in it and sends neuroendocrine signals to modulate the secretion of hormones that regulate digestive processes, such as food intake and nutrient utilization. Insulin (bta04910, bta04911) and glucagon (bta04922) are typical examples of hormones involved in these processes. Insulin influences overall nutrient partitioning, as well as muscle protein accumulation and lipid metabolism in response to feeding [136]. It also has been implicated in feed efficiency regulation in several species, including cattle [17, 120, 136]. Insulin signaling activates several pathways that facilitate glucose uptake in muscle and adipose tissues, increasing energy availability and storage [136]. In turn, glucagon opposes the action of insulin and increases the gluconeogenesis rate, converting hepatic glycogen into circulating glucose [137], thus increasing energy substrate availability for tissues and organs. Furthermore, glucagon is secreted in response to high-protein diets, playing a role in protein synthesis, and its signaling has been identified as a significant pathway for feed efficiency in Nellore cattle [138].

Hormonal pathways involved in growth were also enriched in our study. Growth hormone (GH; bta04935) is a somatotropic axis hormone, the main hormonal axis responsible for growth. This hormone is involved in modulating several growth traits in cattle [139]. In transgenic pigs, it was observed that GH promoted animal growth and improved efficiency and lean meat percentage [140]. The thyroid hormone signaling pathway (bta04918, bta04919) has been recognized to play a key role in muscle formation and fat metabolism, as well as feed efficiency in pigs and cattle [141, 142]. Estrogens (bta04915) are widely known for their involvement in reproductive events; however, they may play a role in different cellular processes that modulate growth [101]. Studies in cattle show that growth feedback in response to estrogens is related to the stimulation of GH secretion by the pituitary [143]. Estrogen can also interact with thyroid hormone-releasing receptors to promote neuroendocrine responses, which affect several functions in the organism, including growth [144]. Furthermore, the energy intake suppression and the increase in basal energy expenditure appear to be modulated by changes in the estrogen signaling level [145].

#### Energetic metabolism

Cellular energy metabolism occurs mainly in mitochondria through oxidative phosphorylation (bta00190), the main process in ATP production that involves the transport of electrons along the electron transport chain (ETC) in the inner mitochondrial membrane [146]. Few studies have explored the relationship between oxidative phosphorylation and feed efficiency, and there is some controversy. For example, studies conducted in cattle have shown that ADP control of oxidative phosphorylation and mitochondrial respiration rate are greater in feed-efficient animals compared to feed-inefficient ones [147]. Similarly, the ruminal epithelium transcriptome profile showed higher expression of genes associated with oxidative phosphorylation in the rumen epithelium of low-RFI beef steers [148]. In contrast, lower feed efficiency Nellore cattle showed overexpression of genes related to oxidative phosphorylation in the rumen epithelium [149]. This is supported by Dorji et al. [150], who reported differentially expressed genes in the blood of high RFI dairy cattle involved in the oxidative phosphorylation pathway, suggesting reduced mitochondrial activity in more efficient animals. Based on these reports, we justify our findings by assuming that more efficient animals may present an improved mitochondrial activity reflecting greater oxidative efficiency in ATP production, thus reducing energy waste (e.g., in ROS and heat production) and improving the nutrients' conversion into ATP.

#### Amino acid, lipid, and carbohydrate metabolism

Amino acid metabolism is involved in growth, protein synthesis, and animals' health. For example, arginine (bta00330, bta00220) regulates nitric oxide synthesis, contributing to improved blood flow and nutrient supply to tissues [151]. In addition, this amino acid influences the expression of lipid-metabolic genes in adipose tissue and skeletal muscle, promoting the growth of lean muscle tissue while reducing fat deposition [152]. Proline (bta00330), an amino acid derived from arginine, is involved in collagen synthesis and tissue repair, contributing to structural development and tissue integrity [153]. Meanwhile, cysteine and methionine (bta00270) are sulfur amino acids that play a role in protein synthesis. Methionine is necessary for eukaryotic proteins' translation, while cysteine ​​contributes to protein structure by forming disulfide bonds [154]. These two amino acids are the main sulfur sources for the body, which in turn can improve performance by increasing bacterial protein synthesis in the rumen (an excellent protein source for muscle synthesis) and improving amino acid balance in ruminants [155].

Lipid metabolism is a biological event involved in feed efficiency regulation mainly through energy metabolism. Fatty acid and steroid biosynthesis (bta00100) are mediated by energy metabolism and indirectly influence feed efficiency [65]. Glycerolipids (bta00564, bta00561), which encompass triglycerides and phospholipids, play roles in cell signaling, membrane dynamics, and energy efficiency [156]. The breakdown of triglycerides into glycerol and free fatty acids provides essential substrates for β-oxidation and energy production, particularly during periods of increased energy demand [157]. In turn, bile acid biosynthesis (bta00120) not only facilitates lipid digestion and absorption but also activates nuclear receptors and cellular signaling pathways that regulate lipid, glucose, and energy metabolism [158].

Dietary carbohydrates are considered the major energy source, providing more than half of the energy requirements for animal maintenance, growth, and production. In ruminants, most of the ingested carbohydrate (such as starch and cellulose) is fermented in the rumen into volatile fatty acids (VFAs), which are then absorbed through the rumen wall [159]. Once absorbed, VFAs are transported to the liver where they are β-oxidized into acetyl-CoA units, molecules that are in turn oxidized in the citrate cycle (TCA; bta00020) for energy production [160]. For its part, pyruvate metabolism (bta00620) acts as an energy substrate in a similar way. Pyruvate is the final product of the glycolytic pathway and sustains the TCA cycle carbon flow. In mitochondria, pyruvate is oxidized to acetyl-CoA and then destined for energy production via TCA [160]. The TCA cycle and pyruvate metabolism pathways have been associated with feed efficiency in studies conducted in different species [58, 161]. These findings highlight the importance of these metabolic pathways in achieving optimal energy balance and enhancing overall productivity.

## Conclusion

The use of imputed WGS data in GWAS analyses enabled the broad identification of regions and candidate genes throughout the genome that regulate the expression of feed efficiency-related traits in Nellore cattle. In general, the candidate genes found are involved in muscle growth, lipid metabolism, response to oxidative stress, nutrient metabolism, neurotransmission, and oxidative phosphorylation. Moreover, enrichment analysis indicated that these genes act in several signaling pathways related to signal transduction, nervous system, endocrine system, energy metabolism, digestive system, and nutrient metabolism. These results provide insights into the molecular mechanisms underlying feed efficiency in Nellore cattle and offer a genetic basis to guide the breeding of efficient animals, ultimately optimizing resource utilization and the profitability of the production system.

## Supplementary Information

Additional file 1: Fig. S1. Quantile–quantileplots of expected versus observed −log10 values for the five evaluated traits:RFI, DMI, FC, FE, and RWG. The red dashed line represents the expected null distribution under no genetic association, and the shaded area indicates the 95% confidence interval. The genomic inflation factorshown in each panel indicates the absence of relevant genomic inflation. Table S1. SNP identification, chromosome, position, *P*-value, region, and gene identification for residual feed intake trait. Table S2. SNP identification, chromosome, position, *P*-value, region, and gene identification for dry matter intake trait. Table S3. SNP identification, chromosome, position, *P*-value, region, and gene identification for feed conversion trait. Table S4. SNP identification, chromosome, position, *P*-value, region, and gene identification for feed efficiency trait. Table S5. SNP identification, chromosome, position, *P*-value, region, and gene identification for residual weight gain trait. Table S6. Functional enrichment results for biological process GO terms of prioritized genes associated with feed efficiency-related traits in Nellore cattle. Table S7. Functional enrichment results for KEGG pathways of prioritized genes associated with feed efficiency-related traits in Nellore cattle.

## Data Availability

The sequencing data that support the findings of this study are available from Gensys breeding program (https://gensys.com.br/), while the phenotypic data were provided by the Instituto de Zootecnia breeding program (http://www.iz.sp.gov.br/); however, their availability is restricted, as they were used under license for the present study and therefore cannot be publicly released. Nonetheless, the data can be obtained upon reasonable request and with authorization from the programs by contacting the corresponding authors (Dr. Lucia G. Albuquerque: galvao.albuquerque@unesp.br; or Dr. Maria Eugênia Z. Mercadante: mezmercadante@gmail.com).

## Abbreviations

Ach: Acetylcholine
ADG: Average daily gain
ADP: Adenosine diphosphate
*AGRN*: Agrin
Akt: Protein kinase B
*ANKRD1*: Ankyrin repeat domain 1
*ANKRD23*: Ankyrin repeat domain 23
*AREG*: Amphiregulin
*ARG2*: Arginase 2
ATP: Adenosine triphosphate
*B2M*: Beta-2-microglobulin
*BBS2*: Bardet-Biedl syndrome 2
*BMPR1A*: Bone morphogenetic protein receptor type 1A
BTA: *Bos taurus* Autosome
BW: Body weight
BW^0.75^: Mid-test metabolic weight
cAMP: Cyclic adenosine monophosphate
CG: Contemporary group
cGMP: Cyclic guanosine monophosphate
*CH25H*: Cholesterol 25-hydroxylase
*CHAT*: Choline O-acetyltransferase
*COX*: Cytochrome C oxidase
DIT: Days in test
DMI: Dry matter intake
EGF: Epidermal growth factor
*EPGN*: Epithelial mitogen
ER: Endoplasmic reticulum
*EREG*: Epiregulin
ETC: Electron transport chain
*FAS*: Fas cell surface death receptor
FC: Feed conversion
FE: Feed efficiency
*FGFR2*: Fibroblast growth factor receptor 2
GEBV: Genomic estimated breeding value
GH: Growth hormone
GIT: Gastrointestinal tract
GSL: Glycosphingolipid
GO: Gene ontology
*GPAT2*: Glycerol-3-phosphate acyltransferase 2
GWAS: Genome-wide association study
*HSPA5*: Heat shock protein family A (Hsp70) member 5
*HSPB1*: Heat shock protein family B (small) member 1
*HTR7*: 5-Hydroxytryptamine receptor 7
HWE: Hardy-Weinberg equilibrium
IGF: Insulin-like growth factor
*ITPR2*: Inositol 1,4,5-trisphosphate receptor type 2
KEGG: Kyoto Encyclopedia of Genes and Genomes
LD: Linkage disequilibrium
*LIPA*: Lipase A, lysosomal acid type
MAF: Minor allele frequency
MAPK: Mitogen-activated protein kinase
mTOR: Mammalian target of rapamycin
*MUSK*: Muscle associated receptor tyrosine kinase
*MYOF*: Myoferlin
*MYOM2*: Myomesin 2
*PAPPA*: Pappalysin 1
PI3K: Phosphoinositide 3-kinase
PKG: Protein kinase G
*POU2F1*: POU class 2 homeobox 1
QC: Quality control
QTL: Quantitative trait loci
Rap1: Ras-related protein 1
RFI: Residual feed intake
ROS: Reactive oxygen species
RWG: Residual weight gain
SNP: Single nucleotide polymorphism
ssGBLUP: Single-step genomic best unbiased prediction
TCA: Citrate cycle/Tricarboxylic acid cycle
TGF-β: Transforming growth factor beta
*TLR4*: Toll like receptor 4
*TRPA1*: Transient receptor potential cation channel subfamily A member 1
*TXN*: Thioredoxin
*UGCG*: UDP-glucose ceramide glucosyltransferase
VFA: Volatile fatty acid
WGS: Whole-genome sequencing
*WWOX*: WW domain containing oxidoreductase
