# Supplementary material for: Sequence-based genome-wide association study reveals genetic and metabolic mechanisms underlying feed efficiency-related traits in beef cattle
Source: J Anim Sci Biotechnol. 2026 Jan 27;17:16. doi: 10.1186/s40104-025-01341-3 (PMC12837112; doi:10.1186/s40104-025-01341-3)
Supplement: Supplementary file 1 — Additional file 1: Fig. S1. Quantile–quantileplots of expected versus observed −log10 values for the five evaluated traits:RFI, DMI, FC, FE, and RWG. The red dashed line represents the expected null distribution under no genetic association, and the shaded area indicates the 95% confidence interval. The genomic inflation factorshown in each panel indicates the absence of relevant genomic inflation. Table S1. SNP identification, chromosome, position, P-value, region, and gene identification for residual feed intake trait. Table S2. SNP identification, chromosome, position, P-value, region, and gene identification for dry matter intake trait. Table S3. SNP identification, chromosome, position, P-value, region, and gene identification for feed conversion trait. Table S4. SNP identification, chromosome, position, P-value, region, and gene identification for feed efficiency trait. Table S5. SNP identification, chromosome, position, P-value, region, and gene identification for residual weight gain trait. Table S6. Functional enrichment results for biological process GO terms of prioritized genes associated with feed efficiency-related traits in Nellore cattle. Table S7. Functional enrichment results for KEGG pathways of prioritized genes associated with feed efficiency-related traits in Nellore cattle. [file 40104_2025_1341_MOESM1_ESM.docx]

**Supplementary Material -** *Sequence-based genome-wide association study reveals genetic and metabolic mechanisms underlying feed efficiency-related traits in beef cattle*

**List of Figures**

[**Fig. S1 -** Quantile-quantile (QQ) plots. 1](#_Toc214891046)

**List of Tables**

[**Table S1.** SNP identification, chromosome (Chr), position, *P*-value, region (0.25 Mb upstream or downstream from the significant SNP), and gene identification for residual feed intake trait. 2](#_Toc214891047)

[**Table S2.** SNP identification, chromosome (Chr), position, *P*-value, region (0.25 Mb upstream or downstream from the significant SNP), and gene identification for dry matter intake trait. 3](#_Toc214891048)

[**Table S3.** SNP identification, chromosome (Chr), position, *P*-value, region (0.25 Mb upstream or downstream from the significant SNP), and gene identification for feed conversion trait. 4](#_Toc214891049)

[**Table S4.** SNP identification, chromosome (Chr), position, *P*-value, region (0.25 Mb upstream or downstream from the significant SNPs), and gene identification for feed efficiency trait. 7](#_Toc214891050)

[**Table S5.** SNP identification, chromosome (Chr), position, *P*-value, region (0.25 Mb upstream or downstream from the significant SNPs), and gene identification for residual weight gain trait. 7](#_Toc214891051)

[**Table S6.** Functional enrichment results for biological process GO terms of prioritized genes associated with feed efficiency-related traits in Nellore cattle. 8](#_Toc214891052)

[**Table S7.** Functional enrichment results for KEGG pathways of prioritized genes associated with feed efficiency-related traits in Nellore cattle. 10](#_Toc214891053)

**
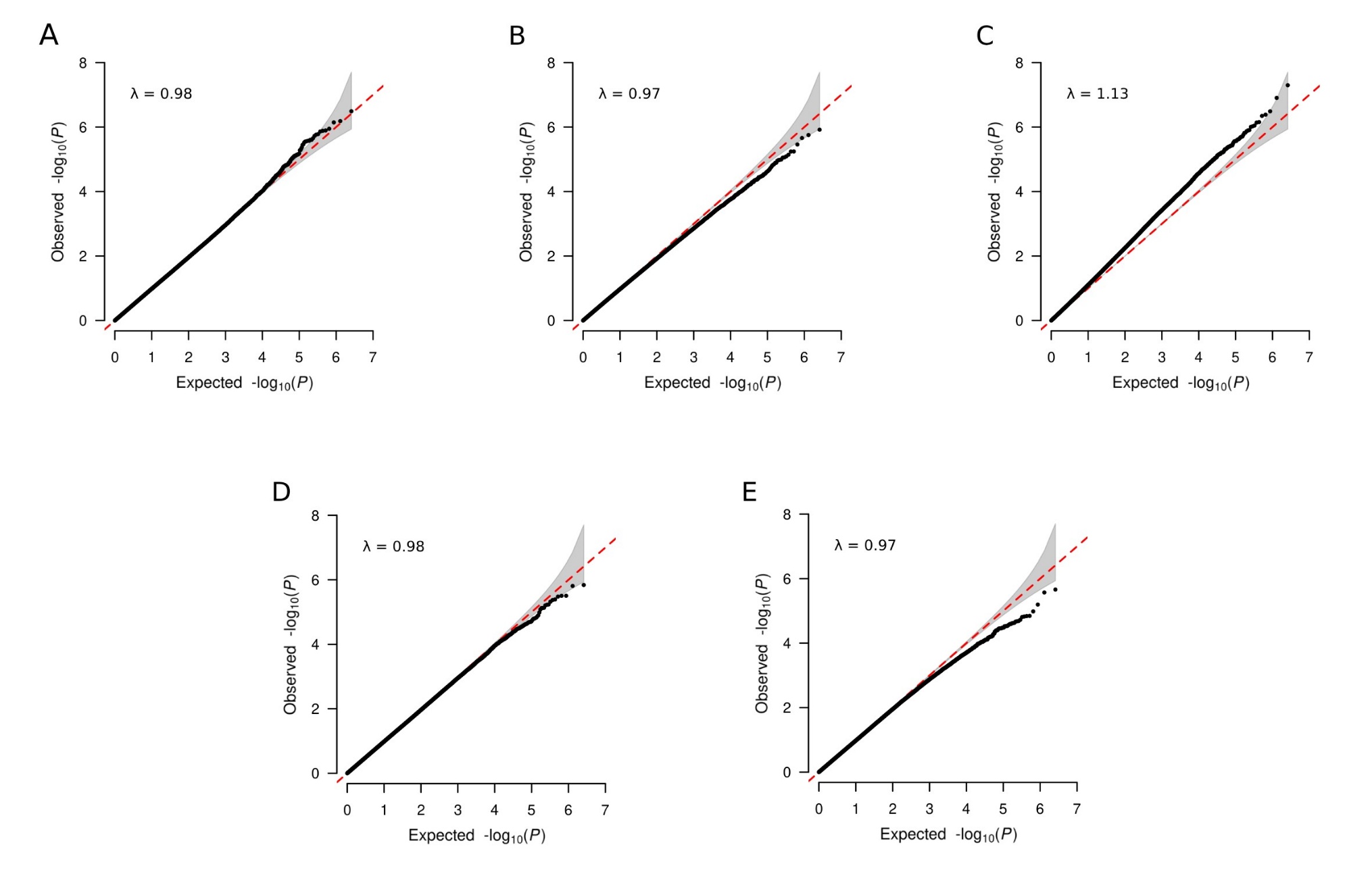
**

**Fig. S1.** Quantile-quantile (QQ) plots of expected versus observed -log_10_(*P*) values for the five evaluated traits: (A) RFI, (B) DMI, (C) FC, (D) FE, and (E) RWG. The red dashed line represents the expected null distribution under no genetic association, and the shaded area indicates the 95% confidence interval. The genomic inflation factor (λ) shown in each panel indicates the absence of relevant genomic inflation.

**Table S1.** SNP identification, chromosome (Chr), position, *P*-value, region (0.25 Mb upstream or downstream from the significant SNP), and gene identification for residual feed intake trait.

| SNP^*^ | Chr | Position (bp) | *P*-value | Region (Mb) | Genes |
| --- | --- | --- | --- | --- | --- |
| rs720977604 | 2 | 129,585,567 | 7.35E-06 | 129.33 – 129.83 | *E2F2, TCEA3, ZNF436, HNRNPR, HTR1D, LUZP1, KDM1A, LOC112443701* |
| rs717406116 | 6 | 10,953,857 | 1.38E-06 | 10.70 – 11.20 | *NDST4, UGT8* |
| rs723737039 | 8 | 99,855,134 | 2.50E-06 | 99.60 – 100.10 | *PALM2, TXN, TXNDC8, SVEP1, LOC112447963* |
| rs719006070 | 8 | 99,984,142 | 8.63E-06 | 99.73 – 100.23 | *TXN, TXNDC8, SVEP1, MUSK* |
| rs380393638 | 8 | 100,012,970 | 4.25E-06 | 99.76 – 100.26 | *TXN, TXNDC8, SVEP1, MUSK* |
| n/a | 8 | 100,026,033 | 6.54E-07 | 99.77 – 100.27 | *TXN, TXNDC8, SVEP1, MUSK* |
| n/a | 8 | 100,026,465 | 1.88E-06 | 99.77 – 100.27 | *TXN, TXNDC8, SVEP1, MUSK* |
| rs716609857 | 8 | 100,158,710 | 3.41E-06 | 99.90 – 100.40 | *SVEP1, MUSK, LPAR1* |
| rs464686822 | 8 | 100,444,249 | 7.17E-06 | 100.19 – 100.69 | *MUSK, LPAR1, SNORA71* |
| rs133003481 | 8 | 100,449,657 | 1.31E-06 | 100.19 – 100.69 | *MUSK, LPAR1, SNORA71* |
| rs457858356 | 8 | 100,503,081 | 2.67E-06 | 100.25 – 100.75 | *MUSK, LPAR1, SNORA71* |
| rs211448414 | 8 | 100,504,299 | 7.98E-06 | 100.25 – 100.75 | *MUSK, LPAR1, SNORA71* |
| rs210300088 | 8 | 100,504,354 | 2.66E-06 | 100.25 – 100.75 | *MUSK, LPAR1, SNORA71* |
| rs207602300 | 8 | 100,504,356 | 3.23E-07 | 100.25 – 100.75 | *MUSK, LPAR1, SNORA71* |
| n/a | 8 | 100,508,809 | 1.66E-06 | 100.25 – 100.75 | *MUSK, LPAR1, SNORA71* |
| rs208165232 | 8 | 100,509,544 | 2.72E-06 | 100.25 – 100.75 | *MUSK, LPAR1, SNORA71* |
| rs210659932 | 8 | 100,509,719 | 9.34E-06 | 100.25 – 100.75 | *MUSK, LPAR1, SNORA71* |
| rs457271459 | 8 | 100,723,515 | 3.00E-06 | 100.47 – 100.97 | *ZNF483, PTGR1, LPAR1, ECPAS, OR2K2, LOC112447938* |
| rs482938768 | 8 | 100,751,060 | 2.50E-06 | 100.50 – 101.00 | *ZNF483, PTGR1, LPAR1, ECPAS, OR2K2, LOC112447938* |
| rs210354997 | 8 | 100,754,351 | 1.27E-06 | 100.50 – 101.00 | *ZNF483, PTGR1, LPAR1, ECPAS, OR2K2, LOC112447938* |
| rs207942236 | 8 | 100,786,243 | 7.17E-07 | 100.53 – 101.03 | *ZNF483, PTGR1, ECPAS, OR2K2, LOC112447938* |
| rs448357616 | 8 | 100,790,072 | 6.88E-06 | 100.54 – 101.04 | *ZNF483, PTGR1, ECPAS, OR2K2, LOC112447938* |
| rs211041233 | 8 | 100,790,214 | 8.09E-06 | 100.54 – 101.04 | *ZNF483, PTGR1, ECPAS, OR2K2, LOC112447938* |
| rs719208928 | 8 | 100,791,904 | 8.76E-06 | 100.54 – 101.04 | *ZNF483, PTGR1, ECPAS, OR2K2, LOC112447938* |
| rs208058118 | 8 | 100,792,091 | 2.84E-06 | 100.54 – 101.04 | *ZNF483, PTGR1, ECPAS, OR2K2, LOC112447938* |
| rs716095978 | 8 | 101,186,674 | 7.11E-06 | 100.93 – 101.43 | *ZNF483, PTGR1, DNAJC25, GNG10, SHOC1, UGCG* |
| rs134403018 | 8 | 101,193,051 | 9.69E-06 | 100.94 – 101.44 | *ZNF483, PTGR1, DNAJC25, GNG10, SHOC1, UGCG, SUSD1* |
| rs714702023 | 8 | 101,388,993 | 7.68E-06 | 101.13 – 101.63 | *SHOC1, UGCG, SUSD1, PTBP3, LOC534155, LOC112447949* |
| rs462307876 | 8 | 101,392,854 | 5.16E-06 | 101.14 – 101.64 | *SHOC1, UGCG, SUSD1, PTBP3, LOC534155, LOC112447949* |
| rs434984939 | 8 | 101,393,971 | 9.53E-06 | 101.14 – 101.64 | *SHOC1, UGCG, SUSD1, PTBP3, LOC534155, LOC112447949* |
| rs479450812 | 8 | 101,399,329 | 7.12E-06 | 101.14 – 101.64 | *SHOC1, UGCG, SUSD1, PTBP3, LOC534155, LOC112447949* |
| rs524607940 | 8 | 101,937,361 | 3.57E-06 | 101.68 – 102.18 | *PTBP3, HSDL2, KIAA1958, INIP, SNX30, SLC46A2, LOC534155* |
| rs524693317 | 8 | 101,937,393 | 2.80E-06 | 101.68 – 102.18 | *KIAA1958, INIP, SNX30, SLC46A2, PTBP3, HSDL2, LOC534155* |
| rs437216580 | 8 | 102,110,693 | 1.71E-06 | 101.86 – 102.36 | *KIAA1958, INIP, SNX30, SLC46A2, ZFP37, LOC513329, LOC783399* |
| rs470743122 | 8 | 102,346,126 | 1.13E-06 | 102.09 – 102.59 | *SNX30, SLC46A2, SLC31A2, FKBP15, SLC31A1, CDC26, PRPF4, RNF183, WDR31, ZFP37, LOC513329, LOC783399* |
| rs716304248 | 8 | 102,346,437 | 2.23E-06 | 102.09 – 102.59 | *SNX30, SLC46A2, SLC31A2, FKBP15, SLC31A1, CDC26, PRPF4, RNF183, WDR31, ZFP37, LOC513329, LOC783399* |
| rs385239653 | 8 | 102,370,025 | 7.67E-06 | 102.12 – 102.62 | *SLC46A2, SLC31A2, FKBP15, SLC31A1, CDC26, PRPF4, RNF183, WDR31, BSPRY, ZFP37, LOC513329, LOC783399* |
| rs41661175 | 8 | 105,505,709 | 5.26E-06 | 105.25 – 105.75 | *PAPPA, ASTN2* |
| rs136854980 | 8 | 108,105,867 | 7.68E-06 | 107.85 – 108.35 | *-* |
| rs210261260 | 8 | 108,132,911 | 4.71E-06 | 107.88 – 108.38 | *-* |
| rs1116420601 | 18 | 23,978,482 | 8.86E-06 | 123.72 – 24.22 | *MMP2, LPCAT2, SLC6A2, CES1, MT2A, GNAO1, BBS2, AMFR, LOC613358, LOC508916, LOC768319* |
| rs384552408 | 26 | 41,463,913 | 6.65E-06 | 41.21 – 41.71 | *FGFR2, ATE1* |
| ^*^ Significant SNP (p < 10^-5^). n/a: not available. | | | | | |

**Table S2.** SNP identification, chromosome (Chr), position, *P*-value, region (0.25 Mb upstream or downstream from the significant SNP), and gene identification for dry matter intake trait.

| SNP^*^ | Chr | Position (pb) | *P*-value | Region (Mb) | Genes |
| --- | --- | --- | --- | --- | --- |
| rs470541724 | 3 | 37,996,454 | 8.12E-06 | 37.74 - 38.24 | *-* |
| rs209250560 | 3 | 40,101,876 | 1.21E-06 | 39.85 - 40.35 | *RNPC3, COL11A1* |
| rs475982324 | 5 | 83,295,165 | 8.72E-06 | 83.04 - 83.54 | *ITPR2* |
| rs207894435 | 6 | 89,235,754 | 7.22E-06 | 88.98 - 89.48 | *CXCL3, GRO1, MGSA, MTHFD2L, EPGN, EREG, AREG* |
| rs134799569 | 8 | 107,258,644 | 8.92E-06 | 107.00 - 107.50 | *TLR4* |
| rs109910904 | 8 | 108,127,842 | 2.19E-06 | 107.87 - 108.37 | *LOC112447940* |
| rs210261260 | 8 | 108,132,911 | 3.48E-06 | 107.88 - 108.38 | *LOC112447940* |
| rs134401789 | 12 | 59,037,132 | 1.77E-06 | 58.78 - 59.28 | *SLITRK1* |
| rs134183878 | 12 | 59,042,554 | 5.73E-06 | 58.79 - 59.29 | *SLITRK1* |
| rs42084340 | 26 | 16,807,020 | 5.71E-06 | 16.55 - 17.05 | *PDLIM1, SORBS1* |

^*^ Significant SNP (p < 10^-5^). n/a: not available.

**Table S3.** SNP identification, chromosome (Chr), position, *P*-value, region (0.25 Mb upstream or downstream from the significant SNP), and gene identification for feed conversion trait.

| SNP^*^ | Chr | Position (pb) | *P*-value | Region (Mb) | | | Genes |
| --- | --- | --- | --- | --- | --- | --- | --- |
| rs717707508 | 1 | 63,992,418 | 2.21E-06 | 63.97 | - | 64.02 | *ARHGAP31, TMEM39A, B4GALT4, UPK1B, IGSF11* |
| rs470969004 | 1 | 64,925,254 | 9.66E-06 | 64.90 | - | 64.95 | *GPR156, FSTL1, LRRC58, GSK3B* |
| rs42683396 | 1 | 99,136,903 | 6.81E-06 | 99.11 | - | 99.16 | *GOLIM4* |
| rs136743165 | 1 | 146,054,476 | 8.96E-06 | 146.03 | - | 146.08 | *MCM3AP, S100B, DIP2A, LSS, COL6A2, FTCD, SPATC1L, PCNT* |
| rs382390574 | 2 | 22,911,840 | 7.47E-06 | 22.89 | - | 22.94 | *SP3, OLA1* |
| rs208131235 | 3 | 2,835,667 | 4.53E-07 | 2.81 | - | 2.86 | *UCK2* |
| rs133889028 | 3 | 2,863,087 | 3.77E-06 | 2.84 | - | 2.89 | *TMCO1, UCK2* |
| rs380167140 | 3 | 2,884,147 | 9.47E-07 | 2.86 | - | 2.91 | *TMCO1, UCK2* |
| rs381621956 | 3 | 2,902,843 | 8.33E-06 | 2.88 | - | 2.93 | *TMCO1, UCK3* |
| rs109732019 | 3 | 63,683,260 | 7.30E-06 | 63.66 | - | 63.71 | *-* |
| rs471150922 | 6 | 96,523,584 | 3.43E-06 | 96.50 | - | 96.55 | *-* |
| rs723204675 | 6 | 96,558,518 | 4.66E-06 | 96.53 | - | 96.58 | *-* |
| rs208174141 | 7 | 32,936,251 | 9.50E-06 | 32.91 | - | 32.96 | *FAM170A* |
| n/a | 7 | 32,942,974 | 7.18E-06 | 32.92 | - | 32.97 | *FAM170A* |
| rs1114466475 | 7 | 33,707,507 | 4.92E-06 | 33.68 | - | 33.73 | *PRR16* |
| rs722390836 | 7 | 33,794,660 | 7.50E-06 | 33.77 | - | 33.82 | *-* |
| rs479930488 | 7 | 35,077,104 | 8.65E-06 | 35.05 | - | 35.10 | *DTWD2, LOC112447624* |
| n/a | 7 | 61,658,318 | 2.73E-06 | 61.63 | - | 61.68 | *SLC6A7, NDST1, PDGFRB, RPS14, CDX1, CSF1R, ARSI, TCOF1, CD74, CAMK2A* |
| rs135589569 | 7 | 71,123,959 | 8.92E-06 | 71.10 | - | 71.15 | *IL12B* |
| rs43641656 | 10 | 73,489,665 | 7.11E-06 | 73.46 | - | 73.51 | *PRKCH, TMEM30B* |
| rs436943216 | 10 | 78,499,204 | 6.11E-06 | 78.47 | - | 78.52 | *GPHN, CCDC196* |
| rs210160193 | 10 | 78,518,549 | 4.88E-06 | 78.49 | - | 78.54 | *GPHN, CCDC197* |
| rs721777275 | 10 | 79,590,415 | 7.02E-07 | 79.57 | - | 79.62 | *ARG2, VTI1B, RDH11,RDH12, TMEM229B, ZFYVE26, PLEKHH1, PIGH, RAD51B* |
| rs210142066 | 10 | 79,600,851 | 5.24E-06 | 79.58 | - | 79.63 | *ARG2, VTI1B, RDH11,RDH12, TMEM229B, ZFYVE26, PLEKHH1, PIGH, RAD51B* |
| rs522867338 | 10 | 79,617,860 | 6.31E-06 | 79.59 | - | 79.64 | *ARG2, VTI1B, RDH11,RDH12, TMEM229B, ZFYVE26, PLEKHH1, PIGH, RAD51B* |
| rs43654711 | 11 | 1,844,123 | 1.74E-06 | 1.82 | - | 1.87 | *ZNF514, PROM2, NPHP1, MRPS5, KCNIP3, BUB1, MAL, LOC617833, LOC112448899, TPC3, ZNF892, ZNF2* |
| rs1115202957 | 11 | 2,521,132 | 2.90E-06 | 2.50 | - | 2.55 | *SNRNP200, NEURL3, FER1L5, LMAN2L, KANSL3, CNNM3, NCAPH, ARID5A, CIAO1, TMEM127, STARD7, CNNM4, ASTL, DUSP2, ITPRIPL1, LOC781566* |
| rs526767012 | 11 | 2,578,072 | 8.55E-06 | 2.55 | - | 2.60 | *SNRNP200, NEURL3, FER1L5, LMAN2L, KANSL3, CNNM3, ANKRD23, NCAPH, ARID5A, CIAO1, TMEM127, STARD7, CNNM4, ITPRIPL1, SEMA4C, ANKRD39, LOC781566* |
| rs723046247 | 11 | 2,624,056 | 1.34E-06 | 2.60 | - | 2.65 | *SNRNP200, NEURL3, FER1L5, LMAN2L, KANSL3, CNNM3, ANKRD23, NCAPH, ARID5A, CIAO1, CNNM4, ITPRIPL1, SEMA4C, ANKRD39, FAM178B, LOC781566* |
| rs718765143 | 11 | 2,627,230 | 4.13E-06 | 2.60 | - | 2.65 | *SNRNP200, NEURL3, FER1L5, LMAN2L, KANSL3, CNNM3, ANKRD23, NCAPH, ARID5A, CNNM4, ITPRIPL1, SEMA4C, ANKRD39, FAM178B, LOC781566* |
| rs522938904 | 11 | 2,766,897 | 4.79E-06 | 2.74 | - | 2.79 | *NEURL3, FER1L5, LMAN2L, KANSL3, CNNM3, ANKRD23, ARID5A, CNNM4, SEMA4C, ANKRD39, ACTR1B, FAM178B, COX5B, LOC112443356* |
| rs437291780 | 11 | 2,767,640 | 3.25E-07 | 2.74 | - | 2.79 | *NEURL3, FER1L5, LMAN2L, KANSL3, CNNM3, ANKRD23, ARID5A, CNNM4, SEMA4C, ANKRD39, ACTR1B, FAM178B, COX5B* |
| rs520557823 | 11 | 2,772,102 | 9.89E-07 | 2.75 | - | 2.80 | *NEURL3, FER1L5, LMAN2L, KANSL3, CNNM3, ANKRD23, ARID5A, CNNM4, SEMA4C, ANKRD39, ACTR1B, FAM178B, COX5B* |
| rs519829296 | 11 | 2,786,569 | 6.86E-06 | 2.76 | - | 2.81 | *FER1L5, LMAN2L, KANSL3, CNNM3, ANKRD23, ARID5A, CNNM4, SEMA4C, ANKRD39, ACTR1B, FAM178B, COX5B* |
| rs521681799 | 11 | 2,789,566 | 7.11E-06 | 2.76 | - | 2.81 | *FER1L5, LMAN2L, KANSL3, CNNM3, ANKRD23, ARID5A, CNNM4, SEMA4C, ANKRD39, ACTR1B, FAM178B, COX5B* |
| rs208742760 | 11 | 2,870,385 | 5.80E-06 | 2.85 | - | 2.90 | *FER1L5, LMAN2L, KANSL3, ZAP70, TMEM131, CNNM3, ANKRD23, CNNM4, SEMA4C, ANKRD39, ACTR1B, FAM178B, COX5B* |
| rs43769490 | 11 | 2,951,107 | 7.28E-06 | 2.93 | - | 2.98 | *LMAN2L, ZAP70, TMEM131, CNNM3, ANKRD23, CNNM4, SEMA4C, ANKRD39, ACTR1B, FAM178B, COX5B* |
| rs459806817 | 11 | 2,961,595 | 3.83E-06 | 2.94 | - | 2.99 | *ZAP70, TMEM131, CNNM3, ANKRD23, CNNM4, SEMA4C, ANKRD39, ACTR1B, FAM178B, COX5B* |
| rs482856980 | 11 | 10,716,235 | 6.10E-06 | 10.69 | - | 10.74 | *MOB1A, STAMBP, ALMS1, TPRKB, DGUOK, ACTG2, TET3, DUSP11, BOLA3* |
| rs471089458 | 12 | 66,610,612 | 3.71E-06 | 66.59 | - | 66.64 | *GPC5* |
| rs525532732 | 12 | 66,677,274 | 4.94E-06 | 66.65 | - | 66.70 | *GPC5* |
| rs715837636 | 12 | 66,678,781 | 2.77E-06 | 66.65 | - | 66.70 | *GPC5* |
| rs459929223 | 12 | 66,680,014 | 6.85E-06 | 66.66 | - | 66.71 | *GPC5* |
| rs465283298 | 12 | 66,682,435 | 6.21E-06 | 66.66 | - | 66.71 | *GPC5* |
| rs722716351 | 12 | 66,682,549 | 9.57E-06 | 66.66 | - | 66.71 | *GPC5* |
| rs525659180 | 12 | 66,685,902 | 8.56E-06 | 66.66 | - | 66.71 | *GPC5* |
| rs475103569 | 12 | 77,565,951 | 5.88E-06 | 77.54 | - | 77.59 | *NALCN* |
| rs480115691 | 12 | 77,668,580 | 4.05E-06 | 77.64 | - | 77.69 | *NALCN, ITGBL1* |
| rs524181866 | 12 | 80,424,595 | 1.52E-06 | 80.40 | - | 80.45 | *-* |
| rs468217230 | 14 | 14,099,211 | 6.94E-06 | 14.07 | - | 14.12 | *LRATD2, LOC112449631* |
| rs42266823 | 14 | 14,148,434 | 9.09E-06 | 14.12 | - | 14.17 | *LRATD2, LOC112449631* |
| rs520207837 | 14 | 14,775,202 | 8.90E-06 | 14.75 | - | 14.80 | *NSMCE2, TRIB1* |
| rs516955614 | 15 | 7,601,688 | 5.38E-06 | 7.58 | - | 7.63 | *TRPC6* |
| rs134784474 | 15 | 25,604,551 | 2.47E-06 | 25.58 | - | 25.63 | *CADM1* |
| rs517045791 | 15 | 32,776,643 | 2.01E-06 | 32.75 | - | 32.80 | *-* |
| rs378631898 | 15 | 32,865,436 | 2.72E-06 | 32.84 | - | 32.89 | *-* |
| rs41765557 | 15 | 32,882,557 | 3.84E-06 | 32.86 | - | 32.91 | *-* |
| rs380201142 | 15 | 76,191,855 | 6.30E-06 | 76.17 | - | 76.22 | *PEX16, AMBRA1, MDK, LARGE2, PHF21A, DGKZ* |
| rs719523582 | 16 | 21,491,939 | 7.59E-06 | 21.47 | - | 21.52 | *RRP15, SPATA17, LOC529125* |
| rs526684608 | 16 | 36,105,144 | 1.97E-06 | 36.08 | - | 36.13 | *RGS7, XCL1, XCL2, DPT* |
| rs445936682 | 16 | 51,447,135 | 2.39E-06 | 51.42 | - | 51.47 | *PUSL1, INTS11, CPTP, SAMD11, ACAP3, AGRN, KLHL17, PLEKHN1, PERM1, HES4, ISG15, TNFRSF18, TNFRSF4, UBE2J2, NOC2L, TTLL10, SPEN, ZBTB17, LOC526769, C1QTNF12, B3GALT6, RNF223* |
| rs519307231 | 19 | 61,046,412 | 6.00E-06 | 61.02 | - | 61.07 | *MAP2K6, ABCA5* |
| rs211542455 | 21 | 29,050,778 | 6.85E-06 | 29.03 | - | 29.08 | *PCSK6, TM2D3, TARS3, SNRPA1* |
| rs720075688 | 21 | 66,085,586 | 8.97E-06 | 66.06 | - | 66.11 | *-* |
| rs719746124 | 21 | 66,091,715 | 3.70E-06 | 66.07 | - | 66.12 | *-* |
| rs42076986 | 25 | 34,433,154 | 4.43E-06 | 34.41 | - | 34.46 | *DTX2, SSC4D, ALKBH4, YWHAG, PRKRIP1, POLR2J, RASA4B, SRRM3, STYXL1, MDH2, SH2B2, HSPB1, ORAI2, LRWD1, ZP3, TMEM120A, POR, UPK3BL2, UPK3B, LOC107131841* |
| rs42963285 | 26 | 12,089,444 | 7.83E-06 | 12.06 | - | 12.11 | *-* |
| rs42963256 | 26 | 12,091,004 | 3.09E-06 | 12.07 | - | 12.12 | *-* |
| rs457222177 | 26 | 12,179,999 | 5.65E-06 | 12.15 | - | 12.20 | *HTR7* |
| rs383020269 | 26 | 12,318,682 | 6.54E-06 | 12.29 | - | 12.34 | *RPP30, HTR7, ANKRD1* |
| rs110525039 | 26 | 14,493,268 | 8.54E-06 | 14.47 | - | 14.52 | *MYOF, EXOC6, CYP26A1, CYP26C1* |
| rs456292449 | 26 | 24,921,397 | 4.52E-06 | 24.90 | - | 24.95 | *ITPRIP, CFAP58, CFAP43, GSTO2,* |
| rs724133064 | 26 | 44,000,256 | 1.48E-06 | 43.98 | - | 44.03 | *OAT, LHPP, FAM53B, NKX1-2* |
| rs526870606 | 27 | 33,790 | 6.86E-06 | 0.01 | - | 0.06 | *LOC132344025* |
| n/a | 27 | 81,665 | 4.12E-07 | 0.06 | - | 0.11 | *LOC132344025* |
| n/a | 27 | 82,097 | 2.06E-06 | 0.06 | - | 0.11 | *LOC132344025* |
| n/a | 27 | 83,424 | 1.24E-06 | 0.06 | - | 0.11 | *LOC132344025* |
| n/a | 27 | 88,572 | 4.37E-06 | 0.06 | - | 0.11 | *LOC132344025* |
| rs722296142 | 27 | 92,160 | 1.27E-06 | 0.07 | - | 0.12 | *LOC132344025* |
| rs519595189 | 27 | 159,645 | 9.19E-06 | 0.13 | - | 0.18 | *LOC132344025* |
| n/a | 27 | 227,818 | 4.01E-06 | 0.20 | - | 0.25 | *LOC132344025* |
| rs385037125 | 27 | 278,289 | 9.50E-06 | 0.25 | - | 0.30 | *ERICH1, LOC132344025* |
| rs521751213 | 27 | 297,607 | 7.33E-06 | 0.27 | - | 0.32 | *ERICH1, LOC132344025* |
| n/a | 27 | 382,936 | 4.12E-06 | 0.36 | - | 0.41 | *ERICH1* |
| n/a | 27 | 398,969 | 4.78E-06 | 0.37 | - | 0.42 | *ERICH1* |
| rs716754904 | 27 | 900,745 | 6.39E-06 | 0.88 | - | 0.93 | *-* |
| rs381113773 | 27 | 904,844 | 2.56E-06 | 0.88 | - | 0.93 | *-* |
| rs381500705 | 27 | 947,129 | 6.33E-06 | 0.92 | - | 0.97 | *-* |
| rs465506277 | 27 | 947,868 | 7.25E-07 | 0.92 | - | 0.97 | *-* |
| rs378332409 | 27 | 961,566 | 3.99E-06 | 0.94 | - | 0.99 | *-* |
| rs716934326 | 27 | 1,060,740 | 2.34E-06 | 1.04 | - | 1.09 | *-* |
| rs211541054 | 27 | 1,062,946 | 9.02E-07 | 1.04 | - | 1.09 | *-* |
| rs720638906 | 27 | 1,080,931 | 5.06E-08 | 1.06 | - | 1.11 | *-* |
| rs719558401 | 27 | 1,175,510 | 9.74E-06 | 1.15 | - | 1.20 | *ARHGEF10, KBTBD11* |
| rs440369017 | 27 | 1,474,882 | 4.19E-06 | 1.45 | - | 1.50 | *MYOM2, ARHGEF10, KBTBD11* |
| rs474326168 | 27 | 1,475,231 | 1.25E-07 | 1.45 | - | 1.50 | *MYOM2, ARHGEF10, KBTBD11* |
| rs444085515 | 27 | 31,541,591 | 6.12E-06 | 31.52 | - | 31.57 | *UNC5D* |
| rs516828147 | 28 | 43,706,096 | 9.08E-06 | 43.68 | - | 43.73 | *DRGX, SLC18A3, CHAT, OGDHL, PARG, ERCC6* |
| rs520267673 | 28 | 44,029,309 | 2.71E-06 | 44.00 | - | 44.05 | *ZFAND4, MSMB, TIMM23, CHAT, WASHC2A, MARCHF8, NCOA4, OGDHL, PARG* |

^*^ Significant SNP (p < 10^-5^). n/a: not available.

**Table S4.** SNP identification, chromosome (Chr), position, *P*-value, region (0.25 Mb upstream or downstream from the significant SNPs), and gene identification for feed efficiency trait.

| SNP | Chr | Position (bp) | *P*-value | Region (Mb) | Genes |
| --- | --- | --- | --- | --- | --- |
| rs208533064 | 3 | 1,514,981 | 5.97E-06 | 1.26 - 1.76 | *CD247, POU2F1, STYXL2, GPA33* |
| n/a | 3 | 2,123,578 | 3.13E-06 | 1.87 - 2.37 | *MAEL, ILDR2, TADA1, POGK* |
| rs433596376 | 11 | 2,143,056 | 4.08E-06 | 1.89 - 2.39 | *MRPS5, ZNF514, ZNF2, ZNF892, PROM2, KCNIP3, FAHD2A, GPAT2, ADRA2B, ASTL, DUSP2, STARD7, TMEM127, CIAO1, SNRNP200, LOC617833* |
| rs526860161 | 11 | 96,067,709 | 7.55E-06 | 95.81 - 96.31 | *RPL35, WDR38, ARPC5L, GOLGA1, SCAI, PPP6C, RABEPK, HSPA5, GAPVD1, MAPKAP1* |
| rs110087490 | 13 | 8,439,689 | 7.51E-06 | 8.18 - 8.68 | *MACROD2* |
| rs522601318 | 13 | 8,484,074 | 3.14E-06 | 8.23 - 8.73 | *MACROD2* |
| rs721888541 | 18 | 5,960,468 | 1.45E-06 | 5.71 - 6.21 | *WWOX* |
| rs133339462 | 21 | 65,405,561 | 4.26E-06 | 65.15 - 65.65 | *YY1, SLC25A29, SLC25A47, WARS1, WDR25, BEGAIN, DLK1* |
| rs519595189 | 27 | 159,645 | 8.32E-06 | 0 - 0.41 | *LOC132344025* |
| rs465506277 | 27 | 947,868 | 6.22E-06 | 0.69 - 1.19 | *-* |
| rs526292625 | 27 | 1,469,749 | 4.77E-06 | 1.21 - 1.71 | *CLN8, ARHGEF10, KBTBD11, MYOM2* |
| rs440369017 | 27 | 1,474,882 | 3.33E-06 | 1.22 - 1.72 | *CLN8, ARHGEF10, KBTBD11, MYOM2* |
| rs474326168 | 27 | 1,475,231 | 5.83E-06 | 1.22 - 1.72 | *CLN8, ARHGEF10, KBTBD11, MYOM2* |
| rs717258230 | 27 | 1,478,480 | 1.55E-06 | 1.22 - 1.72 | *CLN8, ARHGEF10, KBTBD11, MYOM2* |
| rs516693868 | 28 | 41,286,210 | 7.40E-06 | 41.03 - 41.53 | *GRID1, WAPL, OPN4, LDB3, BMPR1A* |

^*^ Significant SNP (p < 10^-5^). n/a: not available.

**Table S5.** SNP identification, chromosome (Chr), position, *P*-value, region (0.25 Mb upstream or downstream from the significant SNPs), and gene identification for residual weight gain trait.

| SNP^*^ | Chr | Position (bp) | *P*-value | Region (Mb) | Genes |
| --- | --- | --- | --- | --- | --- |
| rs519694690 | 14 | 35,832,886 | 2.69E-06 | 35.58 - 36.08 | *TRPA1, KCNB2* |
| rs723954684 | 14 | 70,672,589 | 2.18E-06 | 70.42 - 70.92 | *TMEM67, CIBAR1, FAM92A1* |
| rs715905779 | 26 | 10,764,814 | 6.38E-06 | 10.51 - 11.01 | *ANKRD22, STAMBPL1, ACTA2, FAS, CH25H, LIPA* |
| ^*^ Significant SNP (p < 10^-5^). | | | | | |

**Table S6.** Functional enrichment results for biological process GO terms of prioritized genes associated with feed efficiency-related traits in Nellore cattle.

| ID | Description | GeneRatio | RichFactor | FoldEnrichment | zScore | *P*-value | *P*.adjust | q-value | geneID |
| --- | --- | --- | --- | --- | --- | --- | --- | --- | --- |
| GO:0033674 | positive regulation of kinase activity | 8/112 | 0.0964 | 11.3029 | 8.7323 | 5.04E-07 | 5.04E-07 | 3.46E-04 | *FGFR2, MUSK, AREG, EREG, EPGN, PDGFRB, CSF1R, CD74* |
| GO:0051347 | positive regulation of transferase activity | 8/112 | 0.0879 | 10.3093 | 8.2642 | 1.03E-06 | 1.03E-06 | 3.52E-04 | *FGFR2, MUSK, AREG, EREG, EPGN, PDGFRB, CSF1R, CD74* |
| GO:0051338 | regulation of transferase activity | 9/112 | 0.0573 | 6.7224 | 6.6894 | 7.62E-06 | 7.62E-06 | 1.24E-03 | *FGFR2, MUSK, AREG, EREG, EPGN, PDGFRB, CSF1R, CD74, TRIB1* |
| GO:0050731 | positive regulation of peptidyl-tyrosine phosphorylation | 4/112 | 0.2222 | 26.0595 | 9.8664 | 1.40E-05 | 1.40E-05 | 1.24E-03 | *AREG, EREG, EPGN, CD74* |
| GO:0016125 | sterol metabolic process | 5/112 | 0.1282 | 15.0343 | 8.1400 | 1.88E-05 | 1.88E-05 | 1.44E-03 | *CYP26A1, CYP26C1, LSS, LIPA, CH25H* |
| GO:0007088 | regulation of mitotic nuclear division | 4/112 | 0.1379 | 16.1749 | 7.5868 | 1.01E-04 | 1.01E-04 | 4.35E-03 | *AREG, EREG, EPGN, BUB1* |
| GO:0007169 | cell surface receptor protein tyrosine kinase signaling pathway | 9/112 | 0.0413 | 4.8413 | 5.3039 | 1.01E-04 | 1.01E-04 | 4.35E-03 | *FGFR2, MUSK, AREG, EREG, EPGN, PDGFRB, GSK3B, CSF1R, ZAP70* |
| GO:0051785 | positive regulation of nuclear division | 3/112 | 0.2308 | 27.0618 | 8.7186 | 1.62E-04 | 1.62E-04 | 6.55E-03 | *AREG, EREG, EPGN* |
| GO:0006939 | smooth muscle contraction | 3/112 | 0.1765 | 20.6943 | 7.5353 | 3.76E-04 | 3.76E-04 | 1.14E-02 | *ADRA2B, ACTG2, HTR7* |
| GO:0042310 | vasoconstriction | 3/112 | 0.1154 | 13.5309 | 5.9313 | 1.36E-03 | 1.36E-03 | 3.22E-02 | *ADRA2B, ACTG2, HTR7* |
| GO:0010564 | regulation of cell cycle process | 6/112 | 0.0414 | 4.8525 | 4.3260 | 1.50E-03 | 1.50E-03 | 3.42E-02 | *AREG, EREG, EPGN, ALMS1, BUB1, ZFYVE26* |
| GO:0010469 | regulation of signaling receptor activity | 3/112 | 0.0938 | 10.9939 | 5.2492 | 2.50E-03 | 2.50E-03 | 5.36E-02 | *AREG, EREG, EPGN* |
| GO:0009065 | glutamine family amino acid catabolic process | 2/112 | 0.2000 | 23.4536 | 6.5873 | 3.10E-03 | 3.10E-03 | 6.26E-02 | *OAT, ARG2* |
| GO:0051247 | positive regulation of protein metabolic process | 7/112 | 0.0306 | 3.5846 | 3.6592 | 3.44E-03 | 3.44E-03 | 6.34E-02 | *AREG, EREG, EPGN, CD74, TRIB1, MOB1A, FAS* |
| GO:0035296 | regulation of tube diameter | 3/112 | 0.0833 | 9.7723 | 4.8878 | 3.51E-03 | 3.51E-03 | 6.34E-02 | *ADRA2B, ACTG2, HTR7* |
| GO:0015012 | heparan sulfate proteoglycan biosynthetic process | 2/112 | 0.1818 | 21.3214 | 6.2530 | 3.77E-03 | 3.77E-03 | 6.63E-02 | *NDST4, NDST1* |
| GO:0003018 | vascular process in circulatory system | 3/112 | 0.0750 | 8.7951 | 4.5790 | 4.74E-03 | 4.74E-03 | 7.69E-02 | *ADRA2B, ACTG2, HTR7* |
| GO:0140014 | mitotic nuclear division | 4/112 | 0.0500 | 5.8634 | 4.0464 | 4.82E-03 | 4.82E-03 | 7.69E-02 | *AREG, EREG, EPGN, BUB1* |
| GO:0006525 | arginine metabolic process | 2/112 | 0.1538 | 18.0412 | 5.7009 | 5.29E-03 | 5.29E-03 | 8.06E-02 | *OAT, ARG2* |
| GO:0080135 | regulation of cellular response to stress | 3/112 | 0.0625 | 7.3292 | 4.0740 | 7.90E-03 | 7.90E-03 | 1.13E-01 | *CD74, PARG, FAS* |
| GO:1901615 | organic hydroxy compound metabolic process | 5/112 | 0.0342 | 4.0160 | 3.3985 | 8.22E-03 | 8.22E-03 | 1.15E-01 | *CYP26A1, CYP26C1, LSS, LIPA, CH25H* |
| GO:0016126 | sterol biosynthetic process | 2/112 | 0.1176 | 13.7962 | 4.8960 | 9.02E-03 | 9.02E-03 | 1.19E-01 | *LSS, CH25H* |
| GO:0001666 | response to hypoxia | 2/112 | 0.1053 | 12.3440 | 4.5889 | 1.12E-02 | 1.12E-02 | 1.42E-01 | *MMP2, KLHL17* |
| GO:0042026 | protein refolding | 2/112 | 0.1000 | 11.7268 | 4.4521 | 1.24E-02 | 1.24E-02 | 1.47E-01 | *HSPA5, HSPB1* |
| GO:0008284 | positive regulation of cell population proliferation | 4/112 | 0.0364 | 4.2643 | 3.1883 | 1.45E-02 | 1.45E-02 | 1.66E-01 | *FGFR2, AREG, EREG, EPGN* |
| GO:0061061 | muscle structure development | 3/112 | 0.0469 | 5.4969 | 3.3444 | 1.73E-02 | 1.73E-02 | 1.80E-01 | *PDLIM1, LDB3, MYOF* |
| GO:0000271 | polysaccharide biosynthetic process | 2/112 | 0.0833 | 9.7723 | 3.9891 | 1.76E-02 | 1.76E-02 | 1.80E-01 | *NDST4, NDST1* |
| GO:0030968 | endoplasmic reticulum unfolded protein response | 2/112 | 0.0833 | 9.7723 | 3.9891 | 1.76E-02 | 1.76E-02 | 1.80E-01 | *AMFR, HSPA5* |
| GO:0043648 | dicarboxylic acid metabolic process | 2/112 | 0.0690 | 8.0874 | 3.5434 | 2.52E-02 | 2.52E-02 | 2.37E-01 | *MDH2, OAT* |
| GO:0006029 | proteoglycan metabolic process | 2/112 | 0.0645 | 7.5657 | 3.3941 | 2.85E-02 | 2.85E-02 | 2.58E-01 | *NDST4, NDST1* |
| GO:0005976 | polysaccharide metabolic process | 2/112 | 0.0606 | 7.1071 | 3.2576 | 3.20E-02 | 3.20E-02 | 2.86E-01 | *NDST4, NDST1* |
| GO:1903047 | mitotic cell cycle process | 5/112 | 0.0239 | 2.8055 | 2.4400 | 3.32E-02 | 3.32E-02 | 2.92E-01 | *AREG, EREG, EPGN, BUB1, ZFYVE26* |
| GO:1901701 | cellular response to oxygen-containing compound | 4/112 | 0.0278 | 3.2574 | 2.5261 | 3.47E-02 | 3.47E-02 | 2.93E-01 | *GNAO1, CXCL3, GSK3B, AGRN* |
| GO:0000280 | nuclear division | 4/112 | 0.0276 | 3.2350 | 2.5097 | 3.54E-02 | 3.54E-02 | 2.93E-01 | *AREG, EREG, EPGN, BUB1* |
| GO:0015695 | organic cation transport | 2/112 | 0.0571 | 6.7010 | 3.1320 | 3.57E-02 | 3.57E-02 | 2.93E-01 | *SLC6A2, SLC25A29* |
| GO:1902652 | secondary alcohol metabolic process | 2/112 | 0.0571 | 6.7010 | 3.1320 | 3.57E-02 | 3.57E-02 | 2.93E-01 | *LSS, CH25H* |
| GO:1901699 | cellular response to nitrogen compound | 3/112 | 0.0349 | 4.0907 | 2.6668 | 3.71E-02 | 3.71E-02 | 2.93E-01 | *GNAO1, GSK3B, AGRN* |
| GO:0006986 | response to unfolded protein | 2/112 | 0.0556 | 6.5149 | 3.0728 | 3.76E-02 | 3.76E-02 | 2.93E-01 | *AMFR, HSPA5* |
| GO:0009247 | glycolipid biosynthetic process | 2/112 | 0.0556 | 6.5149 | 3.0728 | 3.76E-02 | 3.76E-02 | 2.93E-01 | *UGCG, PIGH* |
| GO:0008283 | cell population proliferation | 5/112 | 0.0223 | 2.6176 | 2.2645 | 4.27E-02 | 4.27E-02 | 3.15E-01 | *FGFR2, AREG, EREG, EPGN, TMEM127* |
| GO:0016064 | immunoglobulin mediated immune response | 2/112 | 0.0500 | 5.8634 | 2.8568 | 4.56E-02 | 4.56E-02 | 3.16E-01 | *SVEP1, IL12B* |
| GO:0170035 | L-amino acid catabolic process | 2/112 | 0.0500 | 5.8634 | 2.8568 | 4.56E-02 | 4.56E-02 | 3.16E-01 | *OAT, ARG2* |
| GO:0170040 | proteinogenic amino acid catabolic process | 2/112 | 0.0500 | 5.8634 | 2.8568 | 4.56E-02 | 4.56E-02 | 3.16E-01 | *OAT, ARG2* |
| GO:0080134 | regulation of response to stress | 4/112 | 0.0252 | 2.9501 | 2.2944 | 4.71E-02 | 4.71E-02 | 3.23E-01 | *TLR4, CD74, PARG, FAS* |
| GO:0033554 | cellular response to stress | 8/112 | 0.0169 | 1.9834 | 2.0201 | 4.91E-02 | 4.91E-02 | 3.30E-01 | *AMFR, HSPA5, ERCC6, CD74, PARG, ZFYVE26, NSMCE2, FAS* |

**Table S7.** Functional enrichment results for KEGG pathways of prioritized genes associated with feed efficiency-related traits in Nellore cattle.

| Category | ID | Description | GeneRatio | RichFactor | FoldEnrichment | zScore | *P*-value | *P*.adjust | q-value | geneID |
| --- | --- | --- | --- | --- | --- | --- | --- | --- | --- | --- |
| Amino acid metabolism | bta00330 | Arginine and proline metabolism | 2/87 | 0.0392 | 4.3155 | 2.2734 | 7.82E-02 | 7.82E-02 | 3.23E-01 | *OAT, ARG2* |
| Amino acid metabolism | bta00220 | Arginine biosynthesis | 1/87 | 0.0476 | 5.2403 | 1.8627 | 1.75E-01 | 1.75E-01 | 3.89E-01 | *ARG2* |
| Amino acid metabolism | bta00340 | Histidine metabolism | 1/87 | 0.0455 | 5.0021 | 1.7996 | 1.82E-01 | 1.82E-01 | 3.94E-01 | *FTCD* |
| Amino acid metabolism | bta00270 | Cysteine and methionine metabolism | 1/87 | 0.0185 | 2.0379 | 0.7324 | 3.90E-01 | 3.90E-01 | 5.75E-01 | *MDH2* |
| Carbohydrate metabolism | bta00020 | Citrate cycle (TCA cycle) | 1/87 | 0.0323 | 3.5499 | 1.3617 | 2.47E-01 | 2.47E-01 | 4.29E-01 | *MDH2* |
| Carbohydrate metabolism | bta00630 | Glyoxylate and dicarboxylate metabolism | 1/87 | 0.0303 | 3.3347 | 1.2865 | 2.60E-01 | 2.60E-01 | 4.45E-01 | *MDH2* |
| Carbohydrate metabolism | bta00620 | Pyruvate metabolism | 1/87 | 0.0222 | 2.4455 | 0.9307 | 3.38E-01 | 3.38E-01 | 5.25E-01 | *MDH2* |
| Cell growth and death | bta04110 | Cell cycle | 5/87 | 0.0305 | 3.3551 | 2.9131 | 1.66E-02 | 1.66E-02 | 1.55E-01 | *E2F2, GSK3B, BUB1, YWHAG, ZBTB17* |
| Cell growth and death | bta04114 | Oocyte meiosis | 4/87 | 0.0313 | 3.4389 | 2.6601 | 2.90E-02 | 2.90E-02 | 2.09E-01 | *ITPR2, CAMK2A, BUB1, YWHAG* |
| Cell growth and death | bta04218 | Cellular senescence | 3/87 | 0.0183 | 2.0130 | 1.2531 | 1.87E-01 | 1.87E-01 | 4.00E-01 | *E2F2, ITPR2, MAP2K6* |
| Cell growth and death | bta04217 | Necroptosis | 3/87 | 0.0160 | 1.7654 | 1.0123 | 2.41E-01 | 2.41E-01 | 4.29E-01 | *TLR4, CAMK2A, FAS* |
| Cell growth and death | bta04216 | Ferroptosis | 1/87 | 0.0204 | 2.2458 | 0.8372 | 3.61E-01 | 3.61E-01 | 5.46E-01 | *NCOA4* |
| Cell growth and death | bta04210 | Apoptosis | 2/87 | 0.0136 | 1.4972 | 0.5818 | 3.87E-01 | 3.87E-01 | 5.75E-01 | *ITPR2, FAS* |
| Cell growth and death | bta04115 | p53 signaling pathway | 1/87 | 0.0115 | 1.2649 | 0.2377 | 5.50E-01 | 5.50E-01 | 6.15E-01 | *FAS* |
| Cell motility | bta04820 | Cytoskeleton in muscle cells | 8/87 | 0.0345 | 3.7947 | 4.1265 | 1.19E-03 | 1.19E-03 | 1.15E-01 | *COL11A1, PDLIM1, LDB3, MYOM2, ANKRD1, ACTG2, AGRN, ANKRD23* |
| Cell motility | bta04810 | Regulation of actin cytoskeleton | 3/87 | 0.0129 | 1.4169 | 0.6169 | 3.56E-01 | 3.56E-01 | 5.45E-01 | *FGFR2, LPAR1, PDGFRB* |
| Cell motility | bta04814 | Motor proteins | 2/87 | 0.0098 | 1.0736 | 0.1020 | 5.59E-01 | 5.59E-01 | 6.15E-01 | *ACTG2, ACTA2* |
| Cellular community - eukaryotes | bta04540 | Gap junction | 3/87 | 0.0326 | 3.5885 | 2.3889 | 5.11E-02 | 5.11E-02 | 2.69E-01 | *LPAR1, ITPR2, PDGFRB* |
| Cellular community - eukaryotes | bta04550 | Signaling pathways regulating pluripotency of stem cells | 3/87 | 0.0207 | 2.2768 | 1.4835 | 1.45E-01 | 1.45E-01 | 3.77E-01 | *FGFR2, BMPR1A, GSK3B* |
| Cellular community - eukaryotes | bta04510 | Focal adhesion | 2/87 | 0.0098 | 1.0789 | 0.1091 | 5.57E-01 | 5.57E-01 | 6.15E-01 | *PDGFRB, GSK3B* |
| Cellular community - eukaryotes | bta04520 | Adherens junction | 1/87 | 0.0108 | 1.1833 | 0.1701 | 5.74E-01 | 5.74E-01 | 6.22E-01 | *SORBS1* |
| Chromosome | bta03083 | Polycomb repressive complex | 1/87 | 0.0120 | 1.3259 | 0.2855 | 5.33E-01 | 5.33E-01 | 6.15E-01 | *YY1* |
| Chromosome | bta03082 | ATP-dependent chromatin remodeling | 1/87 | 0.0081 | 0.8947 | -0.1126 | 6.77E-01 | 6.77E-01 | 6.69E-01 | *YY1* |
| Circulatory system | bta04270 | Vascular smooth muscle contraction | 4/87 | 0.0282 | 3.0999 | 2.4141 | 4.01E-02 | 4.01E-02 | 2.52E-01 | *ITPR2, ACTG2, PRKCH, ACTA2* |
| Circulatory system | bta04260 | Cardiac muscle contraction | 1/87 | 0.0095 | 1.0481 | 0.0474 | 6.19E-01 | 6.19E-01 | 6.48E-01 | *COX5B* |
| Circulatory system | bta04261 | Adrenergic signaling in cardiomyocytes | 1/87 | 0.0063 | 0.6965 | -0.3684 | 7.66E-01 | 7.66E-01 | 7.35E-01 | *CAMK2A* |
| Development and regeneration | bta04380 | Osteoclast differentiation | 3/87 | 0.0197 | 2.1720 | 1.3947 | 1.60E-01 | 1.60E-01 | 3.86E-01 | *ITPR2, CSF1R, MAP2K6* |
| Development and regeneration | bta04360 | Axon guidance | 3/87 | 0.0166 | 1.8240 | 1.0717 | 2.27E-01 | 2.27E-01 | 4.29E-01 | *GSK3B, CAMK2A, TRPC6* |
| Digestive system | bta04971 | Gastric acid secretion | 2/87 | 0.0263 | 2.8959 | 1.5890 | 1.52E-01 | 1.52E-01 | 3.83E-01 | *ITPR2, CAMK2A* |
| Digestive system | bta04979 | Cholesterol metabolism | 1/87 | 0.0204 | 2.2458 | 0.8372 | 3.61E-01 | 3.61E-01 | 5.46E-01 | *LIPA* |
| Digestive system | bta04978 | Mineral absorption | 1/87 | 0.0161 | 1.7749 | 0.5862 | 4.33E-01 | 4.33E-01 | 5.83E-01 | *SLC31A1* |
| Digestive system | bta04970 | Salivary secretion | 1/87 | 0.0098 | 1.0789 | 0.0767 | 6.08E-01 | 6.08E-01 | 6.43E-01 | *ITPR2* |
| Digestive system | bta04972 | Pancreatic secretion | 1/87 | 0.0097 | 1.0684 | 0.0668 | 6.11E-01 | 6.11E-01 | 6.44E-01 | *ITPR2* |
| Digestive system | bta04974 | Protein digestion and absorption | 1/87 | 0.0078 | 0.8531 | -0.1609 | 6.94E-01 | 6.94E-01 | 6.83E-01 | *COL11A1* |
| Endocrine system | bta04912 | GnRH signaling pathway | 4/87 | 0.0430 | 4.7332 | 3.4643 | 1.01E-02 | 1.01E-02 | 1.55E-01 | *MMP2, ITPR2, CAMK2A, MAP2K6* |
| Endocrine system | bta04916 | Melanogenesis | 3/87 | 0.0291 | 3.2052 | 2.1547 | 6.71E-02 | 6.71E-02 | 2.93E-01 | *GNAO1, GSK3B, CAMK2A* |
| Endocrine system | bta04935 | Growth hormone synthesis, secretion and action | 3/87 | 0.0250 | 2.7511 | 1.8485 | 9.57E-02 | 9.57E-02 | 3.39E-01 | *ITPR2, GSK3B, MAP2K6* |
| Endocrine system | bta04926 | Relaxin signaling pathway | 3/87 | 0.0227 | 2.5010 | 1.6629 | 1.18E-01 | 1.18E-01 | 3.64E-01 | *MMP2, GNAO1, ACTA2* |
| Endocrine system | bta04915 | Estrogen signaling pathway | 3/87 | 0.0216 | 2.3751 | 1.5638 | 1.32E-01 | 1.32E-01 | 3.64E-01 | *MMP2, GNAO1, ITPR2* |
| Endocrine system | bta04910 | Insulin signaling pathway | 3/87 | 0.0214 | 2.3581 | 1.5502 | 1.35E-01 | 1.35E-01 | 3.64E-01 | *SORBS1, GSK3B, SH2B2* |
| Endocrine system | bta04918 | Thyroid hormone synthesis | 2/87 | 0.0263 | 2.8959 | 1.5890 | 1.52E-01 | 1.52E-01 | 3.83E-01 | *ITPR2, HSPA5* |
| Endocrine system | bta04921 | Oxytocin signaling pathway | 3/87 | 0.0192 | 2.1163 | 1.3461 | 1.69E-01 | 1.69E-01 | 3.87E-01 | *GNAO1, ITPR2, CAMK2A* |
| Endocrine system | bta04925 | Aldosterone synthesis and secretion | 2/87 | 0.0208 | 2.2926 | 1.2189 | 2.17E-01 | 2.17E-01 | 4.27E-01 | *ITPR2, CAMK2A* |
| Endocrine system | bta04922 | Glucagon signaling pathway | 2/87 | 0.0194 | 2.1368 | 1.1108 | 2.40E-01 | 2.40E-01 | 4.29E-01 | *ITPR2, CAMK2A* |
| Endocrine system | bta04929 | GnRH secretion | 1/87 | 0.0154 | 1.6930 | 0.5368 | 4.49E-01 | 4.49E-01 | 5.86E-01 | *ITPR2* |
| Endocrine system | bta04927 | Cortisol synthesis and secretion | 1/87 | 0.0152 | 1.6674 | 0.5210 | 4.54E-01 | 4.54E-01 | 5.89E-01 | *ITPR2* |
| Endocrine system | bta04924 | Renin secretion | 1/87 | 0.0137 | 1.5075 | 0.4168 | 4.88E-01 | 4.88E-01 | 6.03E-01 | *ITPR2* |
| Endocrine system | bta03320 | PPAR signaling pathway | 1/87 | 0.0119 | 1.3101 | 0.2733 | 5.37E-01 | 5.37E-01 | 6.15E-01 | *SORBS1* |
| Endocrine system | bta04911 | Insulin secretion | 1/87 | 0.0118 | 1.2947 | 0.2613 | 5.41E-01 | 5.41E-01 | 6.15E-01 | *CAMK2A* |
| Endocrine system | bta04917 | Prolactin signaling pathway | 1/87 | 0.0118 | 1.2947 | 0.2613 | 5.41E-01 | 5.41E-01 | 6.15E-01 | *GSK3B* |
| Endocrine system | bta04914 | Progesterone-mediated oocyte maturation | 1/87 | 0.0106 | 1.1707 | 0.1593 | 5.78E-01 | 5.78E-01 | 6.22E-01 | *BUB1* |
| Endocrine system | bta04928 | Parathyroid hormone synthesis, secretion and action | 1/87 | 0.0087 | 0.9569 | -0.0445 | 6.52E-01 | 6.52E-01 | 6.58E-01 | *ITPR2* |
| Endocrine system | bta04919 | Thyroid hormone signaling pathway | 1/87 | 0.0082 | 0.9020 | -0.1043 | 6.74E-01 | 6.74E-01 | 6.69E-01 | *GSK3B* |
| Energy metabolism | bta00190 | Oxidative phosphorylation | 1/87 | 0.0061 | 0.6710 | -0.4069 | 7.79E-01 | 7.79E-01 | 7.39E-01 | *COX5B* |
| Environmental adaptation | bta04713 | Circadian entrainment | 2/87 | 0.0198 | 2.1791 | 1.1408 | 2.34E-01 | 2.34E-01 | 4.29E-01 | *GNAO1, CAMK2A* |
| Environmental adaptation | bta04714 | Thermogenesis | 3/87 | 0.0115 | 1.2698 | 0.4223 | 4.22E-01 | 4.22E-01 | 5.80E-01 | *KDM1A, SLC25A29, COX5B* |
| Folding, sorting and degradation | bta04141 | Protein processing in endoplasmic reticulum | 3/87 | 0.0171 | 1.8865 | 1.1334 | 2.13E-01 | 2.13E-01 | 4.24E-01 | *AMFR, HSPA5, UBE2J2* |
| Folding, sorting and degradation | bta03060 | Protein export | 1/87 | 0.0323 | 3.5499 | 1.3617 | 2.47E-01 | 2.47E-01 | 4.29E-01 | *HSPA5* |
| Folding, sorting and degradation | bta04120 | Ubiquitin mediated proteolysis | 1/87 | 0.0062 | 0.6835 | -0.3878 | 7.73E-01 | 7.73E-01 | 7.38E-01 | *UBE2J2* |
| Global and overview maps | bta01240 | Biosynthesis of cofactors | 4/87 | 0.0260 | 2.8583 | 2.2263 | 5.14E-02 | 5.14E-02 | 2.69E-01 | *MTHFD2L, RDH12, RDH11, GPHN* |
| Global and overview maps | bta01230 | Biosynthesis of amino acids | 1/87 | 0.0135 | 1.4871 | 0.4028 | 4.92E-01 | 4.92E-01 | 6.03E-01 | *ARG2* |
| Global and overview maps | bta01232 | Nucleotide metabolism | 1/87 | 0.0114 | 1.2505 | 0.2261 | 5.54E-01 | 5.54E-01 | 6.15E-01 | *DGUOK* |
| Global and overview maps | bta01200 | Carbon metabolism | 1/87 | 0.0087 | 0.9569 | -0.0445 | 6.52E-01 | 6.52E-01 | 6.58E-01 | *MDH2* |
| Glycan biosynthesis and metabolism | bta00534 | Glycosaminoglycan biosynthesis - heparan sulfate / heparin | 2/87 | 0.0833 | 9.1705 | 3.8377 | 1.98E-02 | 1.98E-02 | 1.74E-01 | *NDST4, NDST1* |
| Glycan biosynthesis and metabolism | bta00563 | Glycosylphosphatidylinositol (GPI)-anchor biosynthesis | 1/87 | 0.0323 | 3.5499 | 1.3617 | 2.47E-01 | 2.47E-01 | 4.29E-01 | *PIGH* |
| Immune system | bta04658 | Th1 and Th2 cell differentiation | 3/87 | 0.0309 | 3.4035 | 2.2783 | 5.81E-02 | 5.81E-02 | 2.69E-01 | *CD247, IL12B, ZAP70* |
| Immune system | bta04620 | Toll-like receptor signaling pathway | 3/87 | 0.0244 | 2.6840 | 1.8001 | 1.01E-01 | 1.01E-01 | 3.40E-01 | *TLR4, IL12B, MAP2K6* |
| Immune system | bta04621 | NOD-like receptor signaling pathway | 4/87 | 0.0202 | 2.2232 | 1.6654 | 1.06E-01 | 1.06E-01 | 3.43E-01 | *TXN, ITPR2, TLR4, CXCL3* |
| Immune system | bta04660 | T cell receptor signaling pathway | 3/87 | 0.0234 | 2.5792 | 1.7224 | 1.11E-01 | 1.11E-01 | 3.51E-01 | *CD247, GSK3B, ZAP70* |
| Immune system | bta04650 | Natural killer cell mediated cytotoxicity | 3/87 | 0.0189 | 2.0763 | 1.3106 | 1.76E-01 | 1.76E-01 | 3.89E-01 | *CD247, ZAP70, FAS* |
| Immune system | bta04612 | Antigen processing and presentation | 2/87 | 0.0235 | 2.5893 | 1.4094 | 1.81E-01 | 1.81E-01 | 3.94E-01 | *HSPA5, CD74* |
| Immune system | bta04657 | IL-17 signaling pathway | 2/87 | 0.0211 | 2.3168 | 1.2351 | 2.14E-01 | 2.14E-01 | 4.24E-01 | *CXCL3, GSK3B* |
| Immune system | bta04625 | C-type lectin receptor signaling pathway | 2/87 | 0.0189 | 2.0763 | 1.0671 | 2.51E-01 | 2.51E-01 | 4.32E-01 | *ITPR2, IL12B* |
| Immune system | bta04659 | Th17 cell differentiation | 2/87 | 0.0175 | 1.9306 | 0.9572 | 2.78E-01 | 2.78E-01 | 4.58E-01 | *CD247, ZAP70* |
| Immune system | bta04664 | Fc epsilon RI signaling pathway | 1/87 | 0.0141 | 1.5499 | 0.4454 | 4.78E-01 | 4.78E-01 | 6.01E-01 | *MAP2K6* |
| Immune system | bta04062 | Chemokine signaling pathway | 2/87 | 0.0105 | 1.1523 | 0.2036 | 5.21E-01 | 5.21E-01 | 6.15E-01 | *CXCL3, GSK3B* |
| Immune system | bta04662 | B cell receptor signaling pathway | 1/87 | 0.0106 | 1.1707 | 0.1593 | 5.78E-01 | 5.78E-01 | 6.22E-01 | *GSK3B* |
| Immune system | bta04640 | Hematopoietic cell lineage | 1/87 | 0.0091 | 1.0004 | 0.0004 | 6.36E-01 | 6.36E-01 | 6.52E-01 | *CSF1R* |
| Immune system | bta04622 | RIG-I-like receptor signaling pathway | 1/87 | 0.0088 | 0.9739 | -0.0268 | 6.46E-01 | 6.46E-01 | 6.58E-01 | *IL12B* |
| Immune system | bta04670 | Leukocyte transendothelial migration | 1/87 | 0.0085 | 0.9326 | -0.0706 | 6.62E-01 | 6.62E-01 | 6.64E-01 | *MMP2* |
| Immune system | bta04611 | Platelet activation | 1/87 | 0.0081 | 0.8947 | -0.1126 | 6.77E-01 | 6.77E-01 | 6.69E-01 | *ITPR2* |
| Immune system | bta04613 | Neutrophil extracellular trap formation | 1/87 | 0.0041 | 0.4547 | -0.8227 | 8.93E-01 | 8.93E-01 | 8.13E-01 | *TLR4* |
| Lipid metabolism | bta00100 | Steroid biosynthesis | 2/87 | 0.1000 | 11.0046 | 4.2889 | 1.39E-02 | 1.39E-02 | 1.55E-01 | *LSS, LIPA* |
| Lipid metabolism | bta00564 | Glycerophospholipid metabolism | 4/87 | 0.0370 | 4.0758 | 3.0782 | 1.67E-02 | 1.67E-02 | 1.55E-01 | *LPCAT2, GPAT2, DGKZ, CHAT* |
| Lipid metabolism | bta00565 | Ether lipid metabolism | 2/87 | 0.0364 | 4.0017 | 2.1378 | 8.90E-02 | 8.90E-02 | 3.27E-01 | *LPCAT2, UGT8* |
| Lipid metabolism | bta00600 | Sphingolipid metabolism | 2/87 | 0.0345 | 3.7947 | 2.0443 | 9.74E-02 | 9.74E-02 | 3.39E-01 | *UGCG, UGT8* |
| Lipid metabolism | bta00561 | Glycerolipid metabolism | 2/87 | 0.0294 | 3.2366 | 1.7724 | 1.27E-01 | 1.27E-01 | 3.64E-01 | *GPAT2, DGKZ* |
| Lipid metabolism | bta00120 | Primary bile acid biosynthesis | 1/87 | 0.0588 | 6.4733 | 2.1629 | 1.44E-01 | 1.44E-01 | 3.77E-01 | *CH25H* |
| Lipid metabolism | bta00590 | Arachidonic acid metabolism | 1/87 | 0.0118 | 1.2947 | 0.2613 | 5.41E-01 | 5.41E-01 | 6.15E-01 | *PTGR1* |
| Metabolism of cofactors and vitamins | bta00830 | Retinol metabolism | 4/87 | 0.0556 | 6.1137 | 4.1707 | 4.09E-03 | 4.09E-03 | 1.34E-01 | *CYP26A1, RDH12, RDH11, CYP26C1* |
| Metabolism of cofactors and vitamins | bta00670 | One carbon pool by folate | 2/87 | 0.0488 | 5.3681 | 2.6840 | 5.33E-02 | 5.33E-02 | 2.69E-01 | *MTHFD2L, FTCD* |
| Metabolism of cofactors and vitamins | bta00790 | Folate biosynthesis | 1/87 | 0.0263 | 2.8959 | 1.1214 | 2.94E-01 | 2.94E-01 | 4.74E-01 | *GPHN* |
| Nervous system | bta04725 | Cholinergic synapse | 4/87 | 0.0348 | 3.8277 | 2.9213 | 2.05E-02 | 2.05E-02 | 1.74E-01 | *GNAO1, ITPR2, CAMK2A, CHAT* |
| Nervous system | bta04728 | Dopaminergic synapse | 4/87 | 0.0294 | 3.2366 | 2.5156 | 3.51E-02 | 3.51E-02 | 2.36E-01 | *GNAO1, ITPR2, GSK3B, CAMK2A* |
| Nervous system | bta04726 | Serotonergic synapse | 3/87 | 0.0246 | 2.7060 | 1.8161 | 9.93E-02 | 9.93E-02 | 3.39E-01 | *GNAO1, ITPR2, HTR7* |
| Nervous system | bta04722 | Neurotrophin signaling pathway | 3/87 | 0.0240 | 2.6411 | 1.7685 | 1.05E-01 | 1.05E-01 | 3.43E-01 | *GSK3B, CAMK2A, SH2B2* |
| Nervous system | bta04730 | Long-term depression | 2/87 | 0.0317 | 3.4935 | 1.9015 | 1.12E-01 | 1.12E-01 | 3.51E-01 | *GNAO1, ITPR2* |
| Nervous system | bta04720 | Long-term potentiation | 2/87 | 0.0286 | 3.1442 | 1.7241 | 1.33E-01 | 1.33E-01 | 3.64E-01 | *ITPR2, CAMK2A* |
| Nervous system | bta04727 | GABAergic synapse | 2/87 | 0.0220 | 2.4186 | 1.3020 | 2.00E-01 | 2.00E-01 | 4.19E-01 | *GNAO1, GPHN* |
| Nervous system | bta04724 | Glutamatergic synapse | 2/87 | 0.0174 | 1.9138 | 0.9441 | 2.81E-01 | 2.81E-01 | 4.60E-01 | *GNAO1, ITPR2* |
| Nervous system | bta04723 | Retrograde endocannabinoid signaling | 2/87 | 0.0130 | 1.4292 | 0.5141 | 4.10E-01 | 4.10E-01 | 5.80E-01 | *GNAO1, ITPR2* |
| Nervous system | bta04721 | Synaptic vesicle cycle | 1/87 | 0.0125 | 1.3756 | 0.3230 | 5.20E-01 | 5.20E-01 | 6.15E-01 | *SLC6A2* |
| Nucleotide metabolism | bta00230 | Purine metabolism | 1/87 | 0.0075 | 0.8274 | -0.1919 | 7.06E-01 | 7.06E-01 | 6.87E-01 | *DGUOK* |
| Replication and repair | bta03410 | Base excision repair | 1/87 | 0.0227 | 2.5010 | 0.9556 | 3.31E-01 | 3.31E-01 | 5.20E-01 | *PARG* |
| Replication and repair | bta03420 | Nucleotide excision repair | 1/87 | 0.0169 | 1.8652 | 0.6383 | 4.17E-01 | 4.17E-01 | 5.80E-01 | *ERCC6* |
| Sensory system | bta04750 | Inflammatory mediator regulation of TRP channels | 5/87 | 0.0455 | 5.0021 | 4.0426 | 3.20E-03 | 3.20E-03 | 1.34E-01 | *ITPR2, CAMK2A, MAP2K6, PRKCH, TRPA1* |
| Signal transduction | bta04010 | MAPK signaling pathway | 8/87 | 0.0267 | 2.9346 | 3.2601 | 5.79E-03 | 5.79E-03 | 1.41E-01 | *FGFR2, AREG, EREG, HSPB1, PDGFRB, CSF1R, MAP2K6, FAS* |
| Signal transduction | bta04012 | ErbB signaling pathway | 4/87 | 0.0465 | 5.1184 | 3.6738 | 7.67E-03 | 7.67E-03 | 1.55E-01 | *AREG, EREG, GSK3B, CAMK2A* |
| Signal transduction | bta04151 | PI3K-Akt signaling pathway | 9/87 | 0.0230 | 2.5266 | 2.9554 | 9.01E-03 | 9.01E-03 | 1.55E-01 | *FGFR2, LPAR1, TLR4, AREG, EREG, PDGFRB, GSK3B, CSF1R, YWHAG* |
| Signal transduction | bta04015 | Rap1 signaling pathway | 6/87 | 0.0278 | 3.0568 | 2.9279 | 1.37E-02 | 1.37E-02 | 1.55E-01 | *FGFR2, GNAO1, LPAR1, PDGFRB, CSF1R, MAP2K6* |
| Signal transduction | bta04390 | Hippo signaling pathway | 5/87 | 0.0316 | 3.4825 | 3.0130 | 1.44E-02 | 1.44E-02 | 1.55E-01 | *AREG, BMPR1A, GSK3B, MOB1A, YWHAG* |
| Signal transduction | bta04014 | Ras signaling pathway | 5/87 | 0.0202 | 2.2277 | 1.8719 | 7.39E-02 | 7.39E-02 | 3.13E-01 | *FGFR2, PDGFRB, CSF1R, HTR7, ZAP70* |
| Signal transduction | bta04064 | NF-kappa B signaling pathway | 3/87 | 0.0263 | 2.8959 | 1.9501 | 8.51E-02 | 8.51E-02 | 3.23E-01 | *TLR4, CXCL3, ZAP70* |
| Signal transduction | bta04020 | Calcium signaling pathway | 5/87 | 0.0189 | 2.0842 | 1.7106 | 9.20E-02 | 9.20E-02 | 3.32E-01 | *FGFR2, ITPR2, PDGFRB, CAMK2A, HTR7* |
| Signal transduction | bta04668 | TNF signaling pathway | 3/87 | 0.0224 | 2.4637 | 1.6340 | 1.22E-01 | 1.22E-01 | 3.64E-01 | *CXCL3, MAP2K6, FAS* |
| Signal transduction | bta04072 | Phospholipase D signaling pathway | 3/87 | 0.0195 | 2.1438 | 1.3702 | 1.64E-01 | 1.64E-01 | 3.86E-01 | *LPAR1, PDGFRB, DGKZ* |
| Signal transduction | bta04022 | cGMP-PKG signaling pathway | 3/87 | 0.0174 | 1.9194 | 1.1651 | 2.06E-01 | 2.06E-01 | 4.24E-01 | *ITPR2, ADRA2B, TRPC6* |
| Signal transduction | bta04070 | Phosphatidylinositol signaling system | 2/87 | 0.0204 | 2.2458 | 1.1871 | 2.24E-01 | 2.24E-01 | 4.29E-01 | *ITPR2, DGKZ* |
| Signal transduction | bta04392 | Hippo signaling pathway - multiple species | 1/87 | 0.0345 | 3.7947 | 1.4433 | 2.33E-01 | 2.33E-01 | 4.29E-01 | *MOB1A* |
| Signal transduction | bta04066 | HIF-1 signaling pathway | 2/87 | 0.0177 | 1.9477 | 0.9704 | 2.74E-01 | 2.74E-01 | 4.56E-01 | *TLR4, CAMK2A* |
| Signal transduction | bta04371 | Apelin signaling pathway | 2/87 | 0.0140 | 1.5391 | 0.6220 | 3.74E-01 | 3.74E-01 | 5.61E-01 | *ITPR2, ACTA2* |
| Signal transduction | bta04340 | Hedgehog signaling pathway | 1/87 | 0.0175 | 1.9306 | 0.6748 | 4.07E-01 | 4.07E-01 | 5.80E-01 | *GSK3B* |
| Signal transduction | bta04370 | VEGF signaling pathway | 1/87 | 0.0169 | 1.8652 | 0.6383 | 4.17E-01 | 4.17E-01 | 5.80E-01 | *HSPB1* |
| Signal transduction | bta04330 | Notch signaling pathway | 1/87 | 0.0164 | 1.8040 | 0.6033 | 4.28E-01 | 4.28E-01 | 5.83E-01 | *SPEN* |
| Signal transduction | bta04150 | mTOR signaling pathway | 2/87 | 0.0121 | 1.3339 | 0.4143 | 4.44E-01 | 4.44E-01 | 5.86E-01 | *MAPKAP1, GSK3B* |
| Signal transduction | bta04310 | Wnt signaling pathway | 2/87 | 0.0112 | 1.2296 | 0.2969 | 4.86E-01 | 4.86E-01 | 6.03E-01 | *GSK3B, CAMK2A* |
| Signal transduction | bta04630 | JAK-STAT signaling pathway | 2/87 | 0.0092 | 1.0096 | 0.0137 | 5.93E-01 | 5.93E-01 | 6.35E-01 | *PDGFRB, IL12B* |
| Signal transduction | bta04350 | TGF-beta signaling pathway | 1/87 | 0.0092 | 1.0096 | 0.0096 | 6.32E-01 | 6.32E-01 | 6.52E-01 | *BMPR1A* |
| Signal transduction | bta04024 | cAMP signaling pathway | 1/87 | 0.0040 | 0.4384 | -0.8633 | 9.02E-01 | 9.02E-01 | 8.13E-01 | *CAMK2A* |
| Signaling molecules and interaction | bta04060 | Cytokine-cytokine receptor interaction | 5/87 | 0.0147 | 1.6231 | 1.1186 | 1.95E-01 | 1.95E-01 | 4.12E-01 | *CXCL3, BMPR1A, CSF1R, IL12B, FAS* |
| Signaling molecules and interaction | bta04061 | Viral protein interaction with cytokine and cytokine receptor | 2/87 | 0.0213 | 2.3414 | 1.2515 | 2.10E-01 | 2.10E-01 | 4.24E-01 | *CXCL3, CSF1R* |
| Signaling molecules and interaction | bta04512 | ECM-receptor interaction | 1/87 | 0.0112 | 1.2365 | 0.2146 | 5.58E-01 | 5.58E-01 | 6.15E-01 | *AGRN* |
| Signaling molecules and interaction | bta04080 | Neuroactive ligand-receptor interaction | 3/87 | 0.0076 | 0.8379 | -0.3146 | 7.01E-01 | 7.01E-01 | 6.86E-01 | *LPAR1, ADRA2B, HTR7* |
| Signaling molecules and interaction | bta04514 | Cell adhesion molecules | 1/87 | 0.0059 | 0.6512 | -0.4381 | 7.89E-01 | 7.89E-01 | 7.39E-01 | *SLITRK1* |
| Translation | bta00970 | Aminoacyl-tRNA biosynthesis | 1/87 | 0.0154 | 1.6930 | 0.5368 | 4.49E-01 | 4.49E-01 | 5.86E-01 | *WARS1* |
| Transport and catabolism | bta04148 | Efferocytosis | 4/87 | 0.0237 | 2.6046 | 2.0154 | 6.76E-02 | 6.76E-02 | 2.93E-01 | *CAMK2A, ARG2, LIPA, CH25H* |
| Transport and catabolism | bta04137 | Mitophagy - animal | 2/87 | 0.0182 | 2.0008 | 1.0110 | 2.64E-01 | 2.64E-01 | 4.46E-01 | *AMFR, AMBRA1* |
| Transport and catabolism | bta04146 | Peroxisome | 1/87 | 0.0119 | 1.3101 | 0.2733 | 5.37E-01 | 5.37E-01 | 6.15E-01 | *PEX16* |
| Transport and catabolism | bta04142 | Lysosome | 1/87 | 0.0071 | 0.7805 | -0.2515 | 7.27E-01 | 7.27E-01 | 7.04E-01 | *LIPA* |
| Transport and catabolism | bta04145 | Phagosome | 1/87 | 0.0060 | 0.6629 | -0.4195 | 7.83E-01 | 7.83E-01 | 7.39E-01 | *TLR4* |
| Transport and catabolism | bta04140 | Autophagy - animal | 1/87 | 0.0058 | 0.6361 | -0.4625 | 7.97E-01 | 7.97E-01 | 7.42E-01 | *AMBRA1* |
| Transport and catabolism | bta04144 | Endocytosis | 1/87 | 0.0040 | 0.4420 | -0.8544 | 9.00E-01 | 9.00E-01 | 8.13E-01 | *FGFR2* |
| Xenobiotics biodegradation and metabolism | bta00983 | Drug metabolism - other enzymes | 1/87 | 0.0127 | 1.3930 | 0.3359 | 5.15E-01 | 5.15E-01 | 6.15E-01 | *CES1* |
